# MAVS activates TBK1 and IKKε through TRAFs in NEMO dependent and independent manner

**DOI:** 10.1371/journal.ppat.1006720

**Published:** 2017-11-10

**Authors:** Run Fang, Qifei Jiang, Xiang Zhou, Chenguang Wang, Yukun Guan, Jianli Tao, Jianzhong Xi, Ji-Ming Feng, Zhengfan Jiang

**Affiliations:** 1 Key Laboratory of Cell Proliferation and Differentiation of the Ministry of Education, School of Life Sciences, Peking University, Beijing, China; 2 State Key Laboratory of Protein and Plant Gene Research, School of Life Sciences, Peking University, Beijing, China; 3 Peking-Tsinghua Center for Life Sciences, School of Life Sciences, Peking University, Beijing, China; 4 Institute of Molecular Medicine, Peking University, Beijing, China; 5 Department of Comparative Biomedical Sciences, School of Veterinary Medicine, Louisiana State University, Baton Rouge, Louisiana, United States of America; University of Southern California, UNITED STATES

## Abstract

Mitochondrial antiviral-signaling protein (MAVS) transmits signals from RIG-I-like receptors after RNA virus infections. However, the mechanism by which MAVS activates downstream components, such as TBK1 and IKKα/β, is unclear, although previous work suggests the involvement of NEMO or TBK1-binding proteins TANK, NAP1, and SINTBAD. Here, we report that MAVS-mediated innate immune activation is dependent on TRAFs, partially on NEMO, but not on TBK1-binding proteins. MAVS recruited TBK1/IKKε by TRAFs that were pre-associated with TBK1/IKKε via direct interaction between the coiled-coil domain of TRAFs and the SDD domain of TBK1/IKKε. *TRAF2*^*−/−*^*3*^*−/−*^*5*^*−/−*^*6*^*−/−*^ cells completely lost RNA virus responses. TRAFs’ E3 ligase activity was required for NEMO activation by synthesizing ubiquitin chains that bound to NEMO for NF-κB and TBK1/IKKε activation. NEMO-activated IKKα/β were important for TBK1/IKKε activation through IKKα/β-mediated TBK1/IKKε phosphorylation. Moreover, individual TRAFs differently mediated TBK1/IKKε activation and thus fine-tuned antiviral immunity under physiological conditions.

## Author summary

Innate immunity is the first line of defense against virus infection. RIG-I-like receptors (RLRs) recognize various viral RNA from RNA viruses and initiate host antiviral responses to produce type I interferons (IFNs) and other cytokines. RLRs sense distinct types of viruses by sharing a common adaptor protein called mitochondrial antiviral-signaling protein (MAVS). Although it has been well studied how RLRs recruit and activate MAVS upon virus infection, it remains to be elucidated how MAVS activates its downstream components, including kinases TBK1/IKKε and the IKK complex. Here, by using *TANK*^*−/−*^*NAP1*^*−/−*^*SINTBAD*^*−/−*^, *TRAF2*^*−/−*^*3*^*−/−*^*5*^*−/−*^*6*^*−/−*^ and *TRAF2*^*−/−*^*3*^*−/−*^*5*^*−/−*^*6*^*−/−*^*NEMO*^*−/−*^ 293T cells combined with reconstitution experiments, we discovered that MAVS recruited TBK1/IKKε via TRAFs through pre-associated TRAFs-TBK1/IKKε complex. TBK1/IKKε activation required both TRAFs-mediated TBK1 autophosphorylation and TRAFs-NEMO-IKKβ-mediated TBK1 phosphorylation. We demonstrated that TRAFs’ E3 ligase activity was solely required for NEMO and IKKα/β activation. IKKα/β were crucial for both TBK1 and NF-κB activation. Our results thus demonstrated that MAVS activates TBK1/IKKε through TRAFs in both NEMO-dependent and independent manner. Importantly, a minimal amount of IFNs was produced independent of NF-κB activation during virus infection and that individual TRAFs differently mediated TBK1/IKKε activation, thus fine-tuned antiviral immunity under physiological conditions.

## Introduction

The innate immune system is the first line of defense against microbial infection. Germline encoded pattern recognition receptors (PRRs), such as Toll-like receptors (TLRs), RIG-I-like receptors (RLRs) and NOD-like receptors (NLRs) [1], recognize conserved molecular structures known as pathogen-associated molecular patterns (PAMPs) and initiate host antimicrobial response to produce type I interferons (type I-IFNs) and other cytokines.

RIG-I-like receptors (RLRs), including RIG-I, MDA5 and LGP2, detect both viral double-stranded RNA (dsRNA) and single-stranded RNA without the 5’-7-methylguanosine cap in the cytoplasm [2–4]. Both RIG-I and MDA5 contain a DEAD/H-box RNA helicase domain for RNA binding and two N-terminal tandem CARD domains to transmit signal to downstream adapter proteins; unlike RIG-I and MDA5, LGP2 lacking the N-terminal tandem CARD domains is considered an inhibitory or helper factor for the RLR signaling pathway [5–7]. Although RIG-I and MDA5 sense distinct types of viruses [3, 8], they share a common adaptor protein called mitochondrial antiviral-signaling protein (MAVS), also known as CARDIF, IPS-1 or VISA [9–12].

MAVS consists of an N-terminal CARD domain, a proline-rich region preceding a poorly structured middle segment, and a C-terminal transmembrane domain, which localizes MAVS to the mitochondrial outer membrane. Upon binding of dsRNA, RIG-I and MDA5 undergo conformational changes and release the N-terminal tandem CARD domains [13–15]. The CARD domains of RIG-I are modified with lysine-63 (K63) polyubiquitin chains by the E3 ligases TRIM25 and Riplet [16–18]. The exposed CARD domains of RIG-I and MDA5 subsequently bind to unanchored K63 polyubiquitin chains and form oligomers [19, 20]. The latter gain a high capacity of activating MAVS by inducing MAVS polymerization, presumably through CARD-CARD interactions [21–24]. IRF3 is recruited to MAVS polymers through phosphorylation of two conserved serine and threonine clusters in MAVS by IKKs or TBK1 upon virus infection [25]. Phosphorylated MAVS then binds to a positively charged surface of IRF3, thereby recruiting IRF3 for its phosphorylation and activation by TBK1.

Despite the aforementioned progress, it remains to be elucidated how MAVS activates downstream components, including kinases TBK1/IKKε and the IKK complex. Importantly, the function of TRAF proteins in RLR-MAVS pathway is unclear [26]. MAVS harbors three binding motifs for the TRAF members [12, 27–29]. The motif PVQET (143–147) is known to bind TRAF2, TRAF3 and TRAF5, whereas the other two motifs, PGENSE (153–158) and PEENEY (455–460) are predicted to bind TRAF6 [30]. Mutation of all three TRAF binding motifs completely abrogates its ability to activate downstream signaling cascades [12, 27], suggesting that multiple TRAF proteins act downstream of MAVS. However, this inference has yet to be confirmed genetically. Moreover, although the K63-linked polyubiquitin chains are needed for MAVS-mediated IRF3 activation [26], the identity of the E3 ubiquitin ligase remains unknown. Similarly unknown is the mechanism by which NEMO, the regulatory subunit of the IKK complex, facilitates the MAVS-mediated activation of TBK1 [31].

In addition, the mechanism underlying the recruitment of TBK1 to MAVS remains unclear or even controversial. Two models have been proposed so far. One model posits that MAVS recruits TBK1 through the NEMO-TANK-TBK1 complex [31]. However, virus induced IFNβ production has been shown to be unimpaired in TANK deficient cells [32]. The second model suggests that MAVS recruits TBK1 through TBK1 binding proteins NAP1 or SINTBAD [1], however knock-down of these two proteins did not impair type I-IFN production after viral infection [33, 34].

Here, we report that TBK1 was recruited by MAVS via TRAFs, but was not recruited by NEMO or by TBK1-binding proteins TANK-NAP1-SINTBAD. TBK1 activation required both MAVS-TRAFs-mediated TBK1 oligomerization/autophosphorylation and NEMO-IKKβ-mediated TBK1 phosphorylation. Using CRISPR-Cas9-mediated genome editing, we generated triple-deficient (*TANK*^*−/−*^*NAP1*^*−/−*^*SINTBAD*^*−/−*^) 293T cells and found these TBK1-binding proteins were not required for MAVS-TBK1 activation. Instead, TRAFs were absolutely required for TBK1 recruitment and activation. We found that TRAF2, TRAF3, TRAF5 or TRAF6 were differentially and redundantly involved in MAVS-TBK1 activation and that cellular deficiency of four TRAFs (*TRAF2*^*−/−*^*3*^*−/−*^*5*^*−/−*^*6*^*−/−*^) resulted in complete loss of responsiveness to RNA viral infections. Rescue experiment confirmed that each TRAF protein is capable of differentially transmitting signals to activate TBK1. By generating TRAFs-NEMO penta-deficient (*TRAF2*^*−/−*^*3*^*−/−*^*5*^*−/−*^*6*^*−/−*^*NEMO*^*−/−*^) cells, we demonstrated that TRAFs’ E3 ligase activity was solely required for NEMO and IKKα/β activation. Unexpectedly, IKKα/β were found to be crucial for both TBK1 and NF-κB activation. Interestingly, a truncated TRAF protein containing only the coiled-coil and TRAF-C domain was able to activate TBK1. In addition, TRAFs were found to pre-associate with TBK1 and IKKε. *In vitro* protein interaction experiments showed that TRAFs directly interacted with TBK1/IKKε through the coiled-coil domain of TRAFs and the SDD domain of TBK1 and IKKε.

Our results thus unambiguously demonstrated that MAVS activates TBK1/IKKε through TRAFs in both NEMO-dependent and independent manner. Our data also indicated that although NF-κB activation is important for the full induction of type I-IFNs, a minimal amount of IFNs is produced in *NEMO*^*−/−*^ cells without NF-κB activation.

## Results

### TANK/NAP1/SINTBAD are not required for RLR-MAVS pathway

Affinity purification of the TBK1 protein complex led to the co-purification of three TBK1-binding proteins named TANK, SINTBAD and NAP1 [35], which share a conserved TBK1-binding domain (TBD) and interact with TBK1 constitutively [33, 36]. Previous studies suggested that these TBK1-binding proteins may be required for the activation of TBK1 in the innate immune pathways [33, 34, 36]. To elucidate how TBK1 is recruited and activated by MAVS following RNA virus infection, we first sought to test if any of these three proteins might be required. Considering that redundancy may exist among these proteins, we generated TANK, NAP1 and SINTBAD triple deficient cells with CRISPR/Cas9 in 293T cells, which are known to keep the complete RLR pathway and are convenient for gene targeting (S1A–S1D Fig). When infected with Sendai virus (SeV), this triple-deficient cell responded just as well as WT cells in terms of the level of IRF3 phosphorylation and Viperin (an interferon induced gene (ISG)) induction (S1E Fig). Robust induction of IFNβ and various ISGs in the triple deficient cells was detected by RT-PCR (S1F Fig), with the production of type I-IFNs found to be also intact in the triple-knockout (S1G Fig). These results demonstrate that TBK1-binding proteins TANK, NAP1 and SINTBAD are not required for the RLR-MAVS signaling pathway.

### TRAFs are absolutely required for MAVS-mediated signaling

Next, we determined whether MAVS transmited signals through TRAF proteins. There are seven known TRAF members (TRAF1 to 7) in mammals [37]. Except for TRAF7, all TRAFs harbor a C-terminal TRAF homology domain, which is composed of a coiled-coil region and a β-sandwich (TRAF-C). Most TRAFs, with the exception of TRAF1, contain an N-terminal ring finger domain, followed by a variable number of zinc fingers, which empower the E3 ubiquitin ligase activity to the TRAFs. Based on prior work [12, 27–29] and the structural features of TRAFs, we speculated that TRAF2, 3, 5, 6 functioned downstream of MAVS.

**Fig 1:**
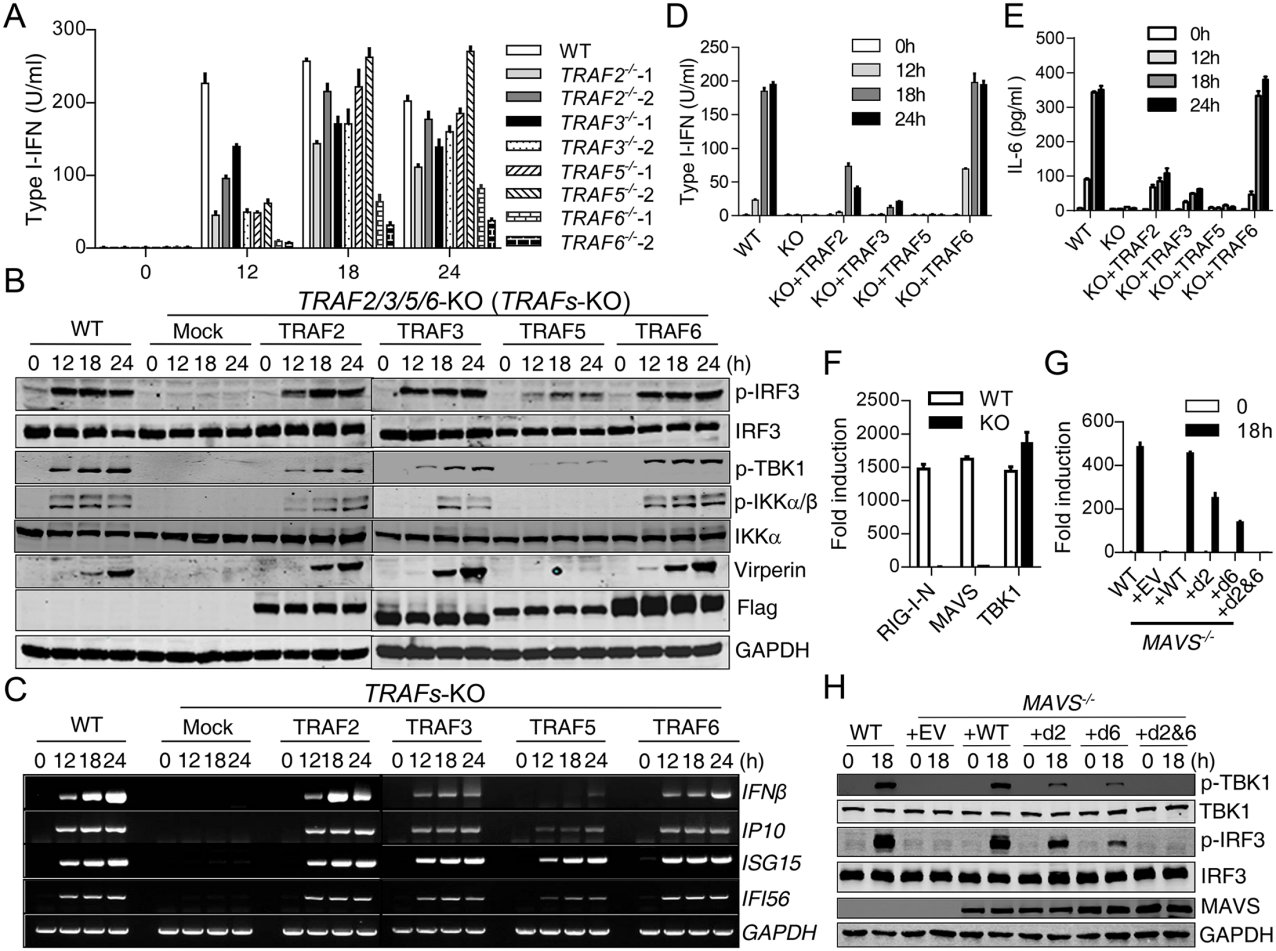
TRAFs are absolutely required for MAVS-mediated signaling. (A) WT and *TRAF2*^−/−^, *TRAF3*^−/−^, *TRAF5*^−/−^, *TRAF6*^−/−^ 293T cells were infected with SeV for the indicated times. Type I-IFN production was determined by bioassay. For each knockout cell, two independent clones were accessed with similar results. (B) to (E) WT 293T cells, 293T cells deficiency of TRAF2, 3, 5, or 6 or cells deficiency of TRAF2, 3, 5, and 6 (TRAFs-deficient) reconstituted with TRAF2, 3, 5, or 6 individually were infected with SeV for the indicated times. Cells were analyzed by Western blot to detect the phosphorylation and expression of the indicated proteins (B), or RT-PCR analysis of the indicated gene induction (C), bioassay analysis of type I-IFN production (D) and ELISA analysis of IL6 production (E). (F) WT and *NEMO*^*−/−*^ 293T cells were transfected with IFNβ–Luc reporter (50 ng) and the indicated plasmids (RIG-I-N 100 ng, MAVS 50 ng, or TBK1 50 ng). Luciferase assay was performed after 24 h. (G) to (H) WT, *MAVS*^−/−^ THP1 cells and *MAVS*^−/−^ THP1 cells reconstituted with MAVS-WT, MAVS-d2 (Q145N), MAVS-d6 (E155D, E457D), or MAVS-d2&6(Q145N, E155D, E457D) were infected with SeV for the indicated times. Type I-IFN production was determined by bioassay (G). Phosphorylation and expression of the indicated proteins were detected by Western blot (H). Data from (A), (D), (E), (F) and (G) represent mean ± SD. Similar results were obtained in 3 independent experiments. See also S2 Fig.

To test which TRAF is critical for MAVS-mediated TBK1 activation, we generated cells deficient in TRAF2, TRAF3, TRAF5 or TRAF6 respectively in 293T cells (S2A–S2D Fig). Only TRAF6-deficient cells showed evident and consistent reduced type I-IFN induction upon SeV infection (Fig 1A). Next, 293T cells deficient of TRAF2, 3, 5, and 6 were generated (each gene targeted with 2 sgRNAs, TRAFs-deficient for short) (S2H Fig) and their responses to virus were tested. As expected, these TRAFs-deficient (*TRAF2*^*−/−*^*3*^*−/−*^*5*^*−/−*^*6*^*−/−*^) cells completely lost their responsivenes to SeV in terms of NF-κB and TBK1-IRF3 activation (Fig 1B), similar to that observed in MAVS-deficient cells. Consistent with this, the induction of type I-IFNs (Fig 1C), ISGs (Fig 1D) and IL-6 (Fig 1E), were all absent in TRAFs-deficient cells. RIG-I-N (the N-terminal CARD module of RIG-I), or MAVS overexpression-induced IFNβ-Luc activation was entirely lost in this TRAFs-deficient cell, whereas TBK1 overexpression caused a comparable induction of IFNβ-Luc compared to the WT cells (Fig 1F). These results indicated that TRAFs were absolutely required for RLR-MAVS activation, and that TRAFs functioned downstream of RIG-I and MAVS, but upstream of TBK1.

Next, we reconstituted this TRAFs-deficient cell with human TRAF2, 3, 5, or 6 respectively and infected each cell with SeV. Consistent with the redundancy of TRAFs, IRF3 phosphorylation was restored in each of the reconstituted cells, as was type I-IFN and ISG induction to some extent (Fig 1B–1D). Among these TRAFs, TRAF6 had the highest ability to re-introduce type I-IFN production, while TRAF5 had the weakest activity. These results collectively suggested that TRAF proteins were absolutely, but redundantly and/or differently, required for MAVS-mediated signaling in cells. TRAF6 was essential and could not be replaced by other TRAF proteins.

**Fig 2:**
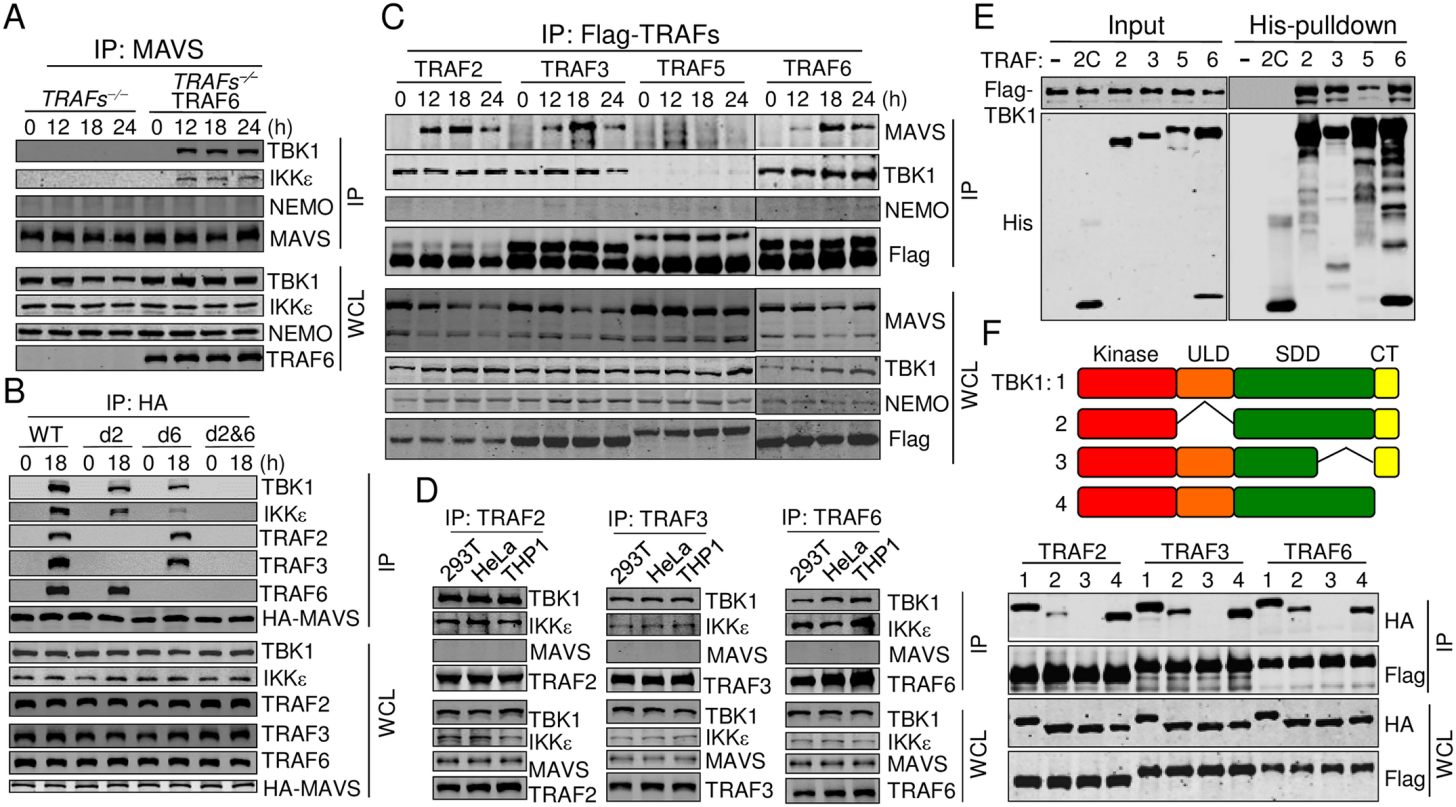
TBK1/IKKε are recruited to MAVS via the pre-associated TRAFs-TBK1/IKKe. (A) 293T cells deficiency of TRAF2, 3, 5, or 6 or TRAFs-deficient cells reconstituted with *TRAF6* were infected with SeV for the indicated times. Cell lysates were immunoprecipitated with the anti-MAVS antibody. The precipitates and whole cell lysates (WCL) were analyzed by Western blot with the indicated antibodies. (B) The reconstituted cells described in Fig 1G were infected with SeV for the indicated times. Cell lysates were immunoprecipitated with the anti-HA antibody. The precipitates and whole cell lysates (WCL) were analyzed by Western blot with the indicated antibodies. (C) The reconstituted cells described in Fig 1B were infected with SeV for the indicated times. Cell lysates were immunoprecipitated with the anti-Flag antibody. The precipitates and whole cell lysates (WCL) were analyzed by Western blot with the indicated antibodies. (D) Cell lysates of 293T, HeLa, and THP1 cells were immunoprecipitated with the anti-TRAF2, 3, or 6 antibodies. The precipitates and whole cell lysates (WCL) were analyzed by Western blot with the indicated antibodies. (E) Recombinant TBK1-Flag and His-tagged TRAFs (TRAF2C: containing amino acids 349–496) were pulled down with Ni-NTA beads. The precipitates and input were analyzed by Western blot with the indicated antibodies. (F) 293T cells were transfected with Flag-tagged TRAFs and full length TBK1 or TBK1 truncations illustrated in the upper panel for 24 h. Cell lysates were immunoprecipitated with the anti-Flag antibody. The precipitates and whole cell lysates (WCL) were analyzed by Western blot with the indicated antibodies. Truncations 1 to 4 indicate full length TBK1 and TBK1 lacking amino acids 309–385, 618–657 and 658–745. See also S3 Fig.

Next, we confirmed the function of TRAFs in MAVS-mediated signaling in the human monocytic THP1 cells by reconstructing MAVS-deficient THP1 cells with the wildtype MAVS or its derived mutants (MAVS-d2 (Q145N), MAVS-d6 (E155D, E457D), MAVS-d2&6 (Q145N, E155D, E457D)). Consistent with previous reports [12, 27–29], MAVS-d2 lost interaction with TRAF2 and TRAF3; MAVS-d6 lost interaction with TRAF6; and MAVS-d2&6 lost interaction with TRAF2, TRAF3 and TRAF6 (Fig 2B). While MAVS-WT completely restored the response of MAVS-deficient THP1 cells to SeV, MAVS-d2&6 did not, confirming that TRAF proteins are also absolutely required for MAVS-mediated activation in THP1 cells (Fig 1G and 1H). Both MAVS-d2 and MAVS-d6 partially restored the response, with MAVS-d2 having a stronger effect than MAVS-d6, confirming that TRAF proteins work redundantly and differentially downstream of MAVS.

### TBK1/IKKε are recruited to MAVS via pre-association with TRAFs

Our results thus far demonstrated that TRAFs were required for the RLR-MAVS pathway. Therefore, we next sought to determine how TRAFs activated TBK1 and IKKε. IKKε is the most closely related paralog of TBK1 and functions indistinguishably to activate IRF3 during RLR-MAVS activation. Given the facts that TRAFs are well known adaptor proteins and that TRAFs function between MAVS and TBK1, we hypothesized that TRAFs may recruit TBK1/IKKε to MAVS, leading to the autophosphorylation and activation of TBK1/IKKε. To test this, MAVS were immunoprecipitated from SeV-infected TRAFs-deficient cells or TRAFs-deficient cells reconstituted with TRAF6. We found that TBK1 and IKKε were recruited to MAVS only in TRAF6-expressing cells upon viral infection (Fig 2A). Consistent with this, whereas MAVS, MAVS-d2, or MAVS-d6 in the reconstituted MAVS-deficient THP1 cells differentially recruited TBK1 or IKKε, MAVS-d2&6 did not (Fig 2B). On the contrary, NEMO was not found to interact with MAVS (Fig 2A). These results indicated that TRAFs were absolutely required for TBK1/IKKε-MAVS interaction and that NEMO did not interact with MAVS.

Next, we analyzed whether TRAFs interacted with MAVS and TBK1. Each TRAF was immunoprecipitated from reconstituted TRAFs-deficient cells after SeV infection and its interaction with TBK1 or MAVS was tested. We found that TRAF2, 3, and 6 but not TRAF5 showed induced interaction with MAVS (Fig 2C), confirming that multiple TRAFs were recruited to MAVS upon infection. Consistent with prior findings, the interaction between TRAF5 and MAVS was too weak to be detected [12]. Interestingly, we found that TBK1 interacted with TRAF2/3/6 constitutively (Fig 2C). Furthermore, NEMO did not directly interact with TRAFs. The constitutive interactions between TRAFs and TBK1/IKKε were confirmed at the endogenous protein level, as TRAF2, TRAF3 or TRAF6 was found to interact vigorously with TBK1/IKKε in non-stimulated 293T, HeLa and THP1 cells (Fig 2D), indicating that the TRAFs-TBK1/IKKε were pre-associated as a complex and recruited to MAVS upon infection. In fact, previous results have demonstrated that TBK1 constitutively interacts with TRAF2, through which TBK1 was identified [38, 39].

We further examined whether TRAFs directly bound to TBK1 using recombinant proteins purified from *E*. *coli*. We found that TRAF2, 3, or 6 had strong interaction with TBK1; TRAF5 again displayed weak but detectable interaction with TBK1; while TRAF2C (TFAF-C domain of TRAF2) had no interaction with TBK1 (Fig 2E). These results strongly indicate that TRAF proteins bind with TBK1 directly.

### TBK1/IKKε-TRAFs interaction is mediated by the TBK1/IKKε SDD domain

TBK1 contains an N-terminal kinase domain (KD), an ubiquitin-like domain (ULD), a scaffold dimerization domain (SDD), and a C-terminal domain (CTD) [40, 41]. To map the domains of TBK1 responsible for TRAF interaction, various deletions of TBK1 were generated and analyzed by co-immunoprecipitation (Fig 2F upper panel). We found that deletion of a segment in the SDD totally abolished its interaction with TRAFs, whereas the ULD deletion had a minor effect (Fig 2F). We also analyzed TRAFs-IKKε interaction and found that IKKε interacted with TRAFs in a very similar way as TBK1. Deletion of the segment of SDD on IKKε wiped out its interaction with TRAFs. However, deletion of IKKε ULD domain did not have any effect (S3A Fig). Based on these results, we concluded that TBK1/IKKε interacted with TRAFs through the SDD domain.

### MAVS activates TBK1/IKKε in a NEMO dependent and independent manner

**Fig 3:**
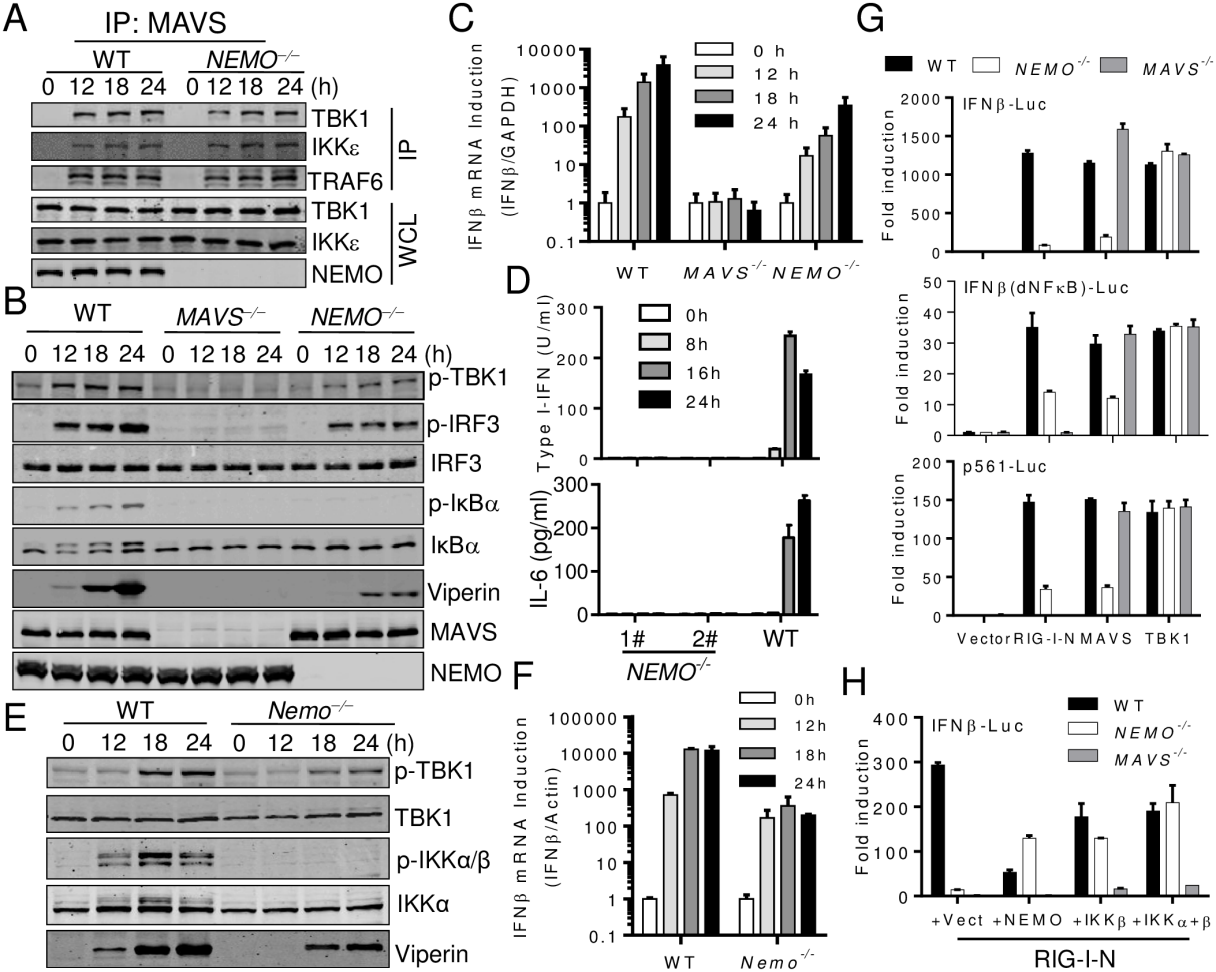
MAVS activates TBK1/IKKε in both NEMO-dependent and independent manner. (A) WT and *NEMO*^−/−^ 293T cells were infected with SeV for the indicated times. Cell lysates were immunoprecipitated with the anti-MAVS antibody. The precipitates and whole cell lysates (WCL) were analyzed by Western blot with the indicated antibodies. (B) and (C) WT, *MAVS*^−/−^ and *NEMO*^−/−^ 293T cells were infected with SeV for the indicated times. Cells were analyzed by Western blot to detect the phosphorylation and expression of the indicated proteins (B), or Quantitative–PCR analysis of *Ifnβ* induction (C). (D) WT and *NEMO*^−/−^ HeLa cells were infected with SeV for the indicated times. Type I-IFN and IL-6 production was determined by bioassay or ELISA respectively. (E) and (F) WT and *Nemo*^−/−^ MEFs were infected with SeV for the indicated times. Cells were analyzed by Western blot to detect the phosphorylation and expression of the indicated proteins (E), or Quantitative–PCR analysis of *Ifnβ* induction (F). (G) WT, *MAVS*^−/−^ and *NEMO*^−/−^ 293T cells were transfected with luciferase reporter constructs (IFNβ–Luc or IFNβ (dNF-κB)-Luc or p561-Luc, 50ng) and the indicated plasmids (empty vector 100 ng, RIG-I-N 100 ng, MAVS 50 ng and TBK1 50 ng). Luciferase assay was performed after 24 h. (H) WT, *MAVS*^−/−^ and *NEMO*^−/−^ 293T cells were transfected with IFNβ–Luc reporter (50 ng) and the indicated plasmids (RIG-I-N 100 ng, empty vector 200ng, NEMO 200 ng, IKKβ 200 ng, IKKα+β 100 ng each). Luciferase assay was performed after 24 h. Data from (C), (D), (F), (G) and (H) represent mean ± SD. Similar results were obtained in 3 independent experiments. See also S4 Fig.

Next, we analyzed how NEMO regulated IRF3/7 activation by generating MAVS-deficient (*MAVS*^*−/−*^) and NEMO-deficient (*NEMO*^*−/−*^) cells (S4A to S4C Fig). First, we tested if NEMO was required for MAVS-TBK1 interaction. We found NEMO deficiency did not have any effects on MAVS-TBK1/IKKε or MAVS-TRAFs interaction (Fig 3A). TBK1/IKKε and TRAFs were recruited to MAVS in *NEMO*^*−/−*^ cells as well as in the wildtype cells. This result confirmed our conclusion that TRAFs recruit TBK1/IKKε to MAVS.

Next, we assessed the activation of various signaling molecules in *MAVS*^*−/−*^ and *NEMO*^*−/−*^ cells. Consistent with previous results, *MAVS*^*−/−*^ cells completely lost their responses to RNA virus. In *NEMO*^*−/−*^ cells, however, only virus-induced IKKα/β and IκBα phosphorylation was lost, whereas diminished but obvious TBK1 and IRF3 phosphorylation was still detected (Fig 3B). Consistently, a minimal induction of type I-IFNs (Fig 3C) and ISGs (S4D Fig) was detected at the mRNA level. However, although the induction of Viperin was evident (Fig 3B), type I-IFNs were not detected by a type I-IFN bioassay due to its low sensitivity (Fig 3D). IL-6 production in *NEMO*^*−/−*^ cells was completely absent. Similar results were obtained from mouse embryonic fibroblasts (MEFs) derived from WT and *Nemo*^*−/−*^ mice (S4E Fig) [42], which were confirmed by Q-PCR and RT-PCR analysis (Figs 3F and S4F). These results collectively indicated that MAVS activated TBK1 in both NEMO-dependent and independent manner. Consistently, RIG-I-N or MAVS overexpression induced IFNβ-Luc (luciferase reporter driven by the IFNβ promoter) induction, IFNβ (dNF-κB)-Luc (IFNβ promoter lacking NF-κB binding sites) induction and p561-Luc (Isg56 promoter only regulated by IRF3) [43] induction were all profoundly impaired in *NEMO*^*−/−*^ 293T cells (Fig 3G), confirming that NEMO was critically involved in supporting TBK1/IKKε-IRF3 activation. Moreover, IKKβ overexpression alone in *NEMO*^*−/−*^ or *MAVS*^*−/−*^ cells activated IFNβ-Luc to some extent (Fig 3H). Since NEMO was required for IKKα/β activation, this result suggested a possible connection between IKKα/β and TBK1/IKKε activation (see below).

### TRAFs’ E3 ligase activity is required for NEMO activation

**Fig 4:**
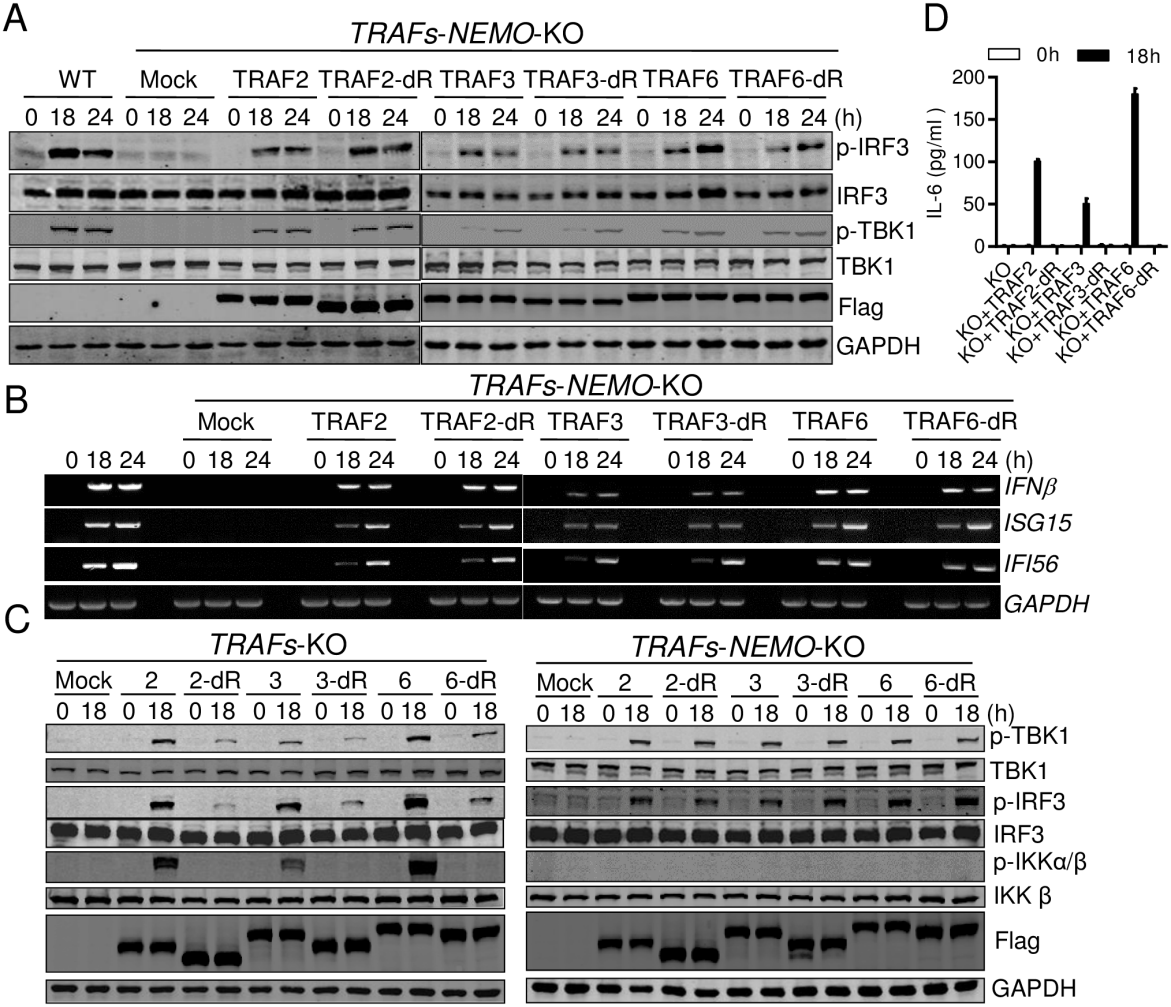
TRAFs’ E3 ligase activity is required for TBK1/IKKε activation via NEMO. (A) and (B) WT 293T cells, 293T cells with combined deficiency of TRAFs and NEMO (TRAFs/NEMO-deficient) and the same deficient cells reconstituted with TRAF2, 3, or 6 or their dRing deletions were infected with SeV for the indicated times. Cells were analyzed by Western blot to detect the phosphorylation and expression of the indicated proteins (A), or RT-PCR analysis of the indicated gene induction (B). (C) WT 293T cells, TRAFs-deficient and TRAFs/NEMO-deficient cells were transiently transfected with TRAF2, 3, or 6 or their dRing deletions for 12 h and infected with SeV for the indicated times. Cells were analyzed by Western blot to detect the phosphorylation and expression of the indicated proteins. (D) Cells in the left panel of (C) were analyzed by ELISA to detect the production of IL6. Data from (D) represent mean ± SD. Similar results were obtained in 3 independent experiments. See also S5 Fig.

Previous studies demonstrated that TRAFs synthesize the K63-linked polyubiquitins to activate NEMO after RNA virus infection [27, 31]. Since we found that NEMO was also critical for TBK1 activation, we wondered whether the E3 ligase activity of TRAFs would be involved in such activation. To exclude the possible involvement of other E3 ligases that may activate NEMO, we deleted NEMO in the TRAFs-deficient cells as well (S5A Fig). When these penta-deficient cells (*TRAF2*^*−/−*^*3*^*−/−*^*5*^*−/−*^*6*^*−/−*^*NEMO*^*−/−*^) were reconstituted with wild-type (WT) TRAF2, 3, 6 or the corresponding dRing mutant of TRAF and followed with SeV infection, we found that all of the reconstituted (WT and dRing) cells showed comparably reduced TBK1 and IRF3 phosphorylation (Fig 4A) and ISG induction (Fig 4B) compared to the wildtype cells, confirming that TRAFs alone could activate TBK1-IRF3 to some extent in the absence of NEMO. This result also suggested that TRAFs’ E3 ubiquitin ligase activity was not required for TBK1 and IRF3 activation at this stage.

On the other side, TRAFs’ Ring domain was absolutely required for MAVS-mediated IKKα/β activation (Fig 4C, left panel). Critically, NEMO functioned downstream of TRAFs for NF-κB activation as TRAFs-NEMO deficient cells showed no NF-κB activation even with the ectopically-expressed TRAFs (Fig 4C, right panel). Moreover, although re-expression of either wildtype-TRAF or dRing-TRAF displayed the same IRF3 phosphorylation in TRAFs-NEMO -deficient cells, re-expression of WT-TRAF in TRAFs-deficient cells showed a much enhanced IRF3 phosphorylation, which was comparable to that in wildtype cells. This result indicated that TRAFs-mediated ubiquitination exerted on NEMO and it was important for the full activation of TBK1. Not surprisingly, reconstitution of the wildtype, but not dRing TRAF, restored SeV infection-induced IL-6 production (Fig 4D).

### TRAFs’ coiled-coil domain is important for both interaction and activation of TBK1/IKKε

**Fig 5:**
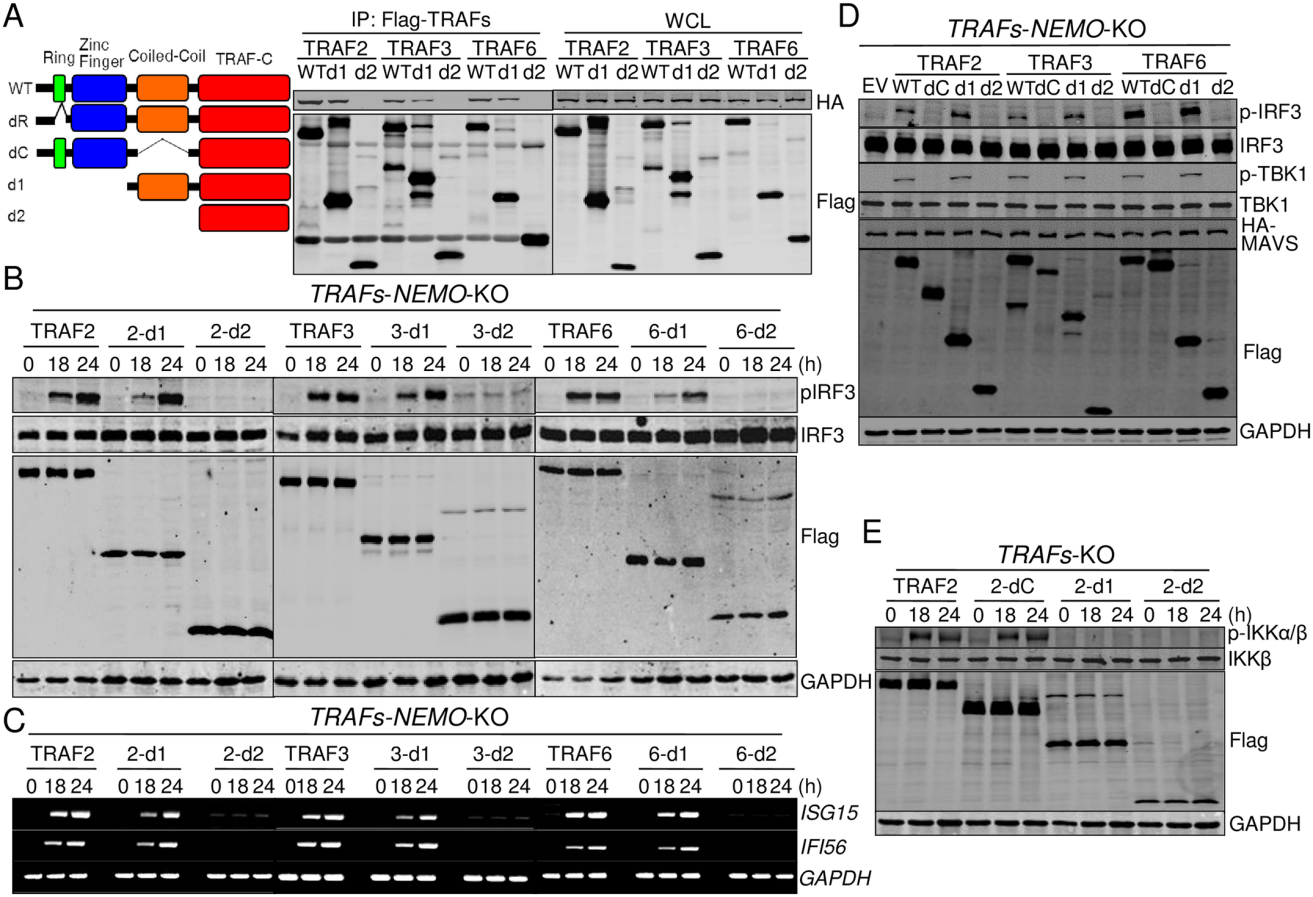
TRAFs’ coiled-coil domain is important for both interaction and activation of TBK1/IKKε. (A) Left: A diagram illustrating TRAF truncations. dR deletions: lacking amino acids 34–73 for TRAF2, 68–77 for TRAF3 and 70–109 for TRAF6; dC deletions: lacking amino acids 299–348 for TRAF2, 267–338 for TRAF3 and 288–348 for TRAF6; d1 deletions: lacking amino acids 1–233 for TRAF2, 1–249 for TRAF3 and 1–259 for TRAF6; d2 deletions: lacking amino acids 1–348 for TRAF2, 1–338 for TRAF3 and 1–347 for TRAF6. Right: 293T cells were transfected with Flag-tagged TRAFs or their truncations and HA-tagged TBK1 for 24 h. Cell lysates were immunoprecipitated with the anti-Flag antibody. The precipitates and whole cell lysates (WCL) were analyzed by Western blot with the indicated antibodies. (B) and (C) TRAFs/NEMO-deficient cells reconstituted with TRAF2, 3, or 6 or their truncations were infected with SeV for the indicated times. Cells were analyzed by Western blot to detect the phosphorylation and expression of the indicated proteins (B), or RT-PCR analysis of the indicated gene induction (C). (D) TRAFs/NEMO-deficient cells were transfected with Flag-tagged TRAFs or their truncations and HA-tagged MAVS for 24 h. Cells were analyzed by Western blot to detect the phosphorylation and expression of the indicated proteins. (E) TRAFs–deficient cells reconstituted with TRAF2 or its truncations were infected with SeV for the indicated times. Cells were analyzed by Western blot to detect the phosphorylation and expression of the indicated proteins.

To determine the domain(s) of TRAFs required for the interaction with TBK1/IKKε, TRAFs deletions were generated and tested by co-immunoprecipitation (Fig 5A). We found that Ring domain deletion had no effect on TRAF-TBK1 interaction. However, this interaction was partially weakened by Ring and Zinc finger deletions (d1) and severely weakened by coiled-coil domain deletions (Figs S6A and 5A), indicating that both Zinc finger and coiled-coil domains of TRAFs were involved. Similar results were observed when TRAFs-IKKε interaction was analyzed (S6B Fig). These deletions of TRAFs did not impair the interaction with MAVS (S6C Fig), confirming that TRAFs interact with MAVS through their C-terminal TRAF domain [44, 45].

Since we found that truncations containing coiled-coil and TRAF-C domains of TRAFs (d1) were the minimum element retaining the interaction with TBK1 and that TRAFs’ E3 ligase activity was only required for NEMO-IKKα/β activation, we deduced that these truncations may also be the minimum region to activate TBK1. To test this, TRAFs-NEMO deficient cells were reconstituted with the aforementioned minimum truncations (d1) or TRAF-C domain (d2), and infected with SeV. Indeed, we found that whereas TRAFs-NEMO-deficient cells reconstituted with d2 truncations did not show any responses to SeV, cells reconstituted with d1 truncations showed partially restored responses to SeV infection as reduced but obvious IRF3 phosphorylation (Fig 5B) and ISG induction (Fig 5C) were detected, which were very similar to those in NEMO-deficient cells. These data confirmed that TRAFs-TBK1 interaction was a prerequisite for TBK1 activation in the RLR pathway and that TRAFs’ E3 ligase activity was required for the full activation of TBK1 and IRF3 via NEMO. Moreover, co-overexpression of MAVS with TRAF2/3/6-WT or TRAF2/3/6-d1, but not with TRAF-dC or TRAF-d2 induced a prominent phosphorylation of TBK1 and IRF3 in TRAFs-NEMO deficient cells (Fig 5D), confirming that the MAVS-TRAF-d1 complex was able to activate TBK1 in the absence of NEMO and that TBK1 might be activated by MAVS-TRAF-TBK1 oligomerization through self-phosphorylation.

Our above data showed that TRAFs’ E3 ligase activity was only required for NEMO activation, which is dependent on the Ring and the Zinc-finger domains of TRAF protein. Since we found that TRAF-d1 truncations supported partial TBK1 activation in TRAFs-NEMO-deficient cells, we next asked if TRAF-dC mutants that lost the coiled-coil domain for TBK1 recruitment would be able to support RLR-MAVS-induced NEMO-NF-κB activation, since this truncated protein harbors MAVS-interacting domain and E3 ligase activity. TRAF2-WT, TRAF2-dC, TRAF2-d1 or TRAF2-d2 were re-introduced respectively in the TRAFs-deficient cells that followed by SeV infection. We found that TRAF2-WT or TRAF2-dC, but not TRAF2-d1 or TRAF2-d2 were able to activate IKKα/β in TRAFs-deficient cells (Fig 5E).

### NEMO is activated through the binding of ubiquitin chains synthetized by TRAFs

**Fig 6:**
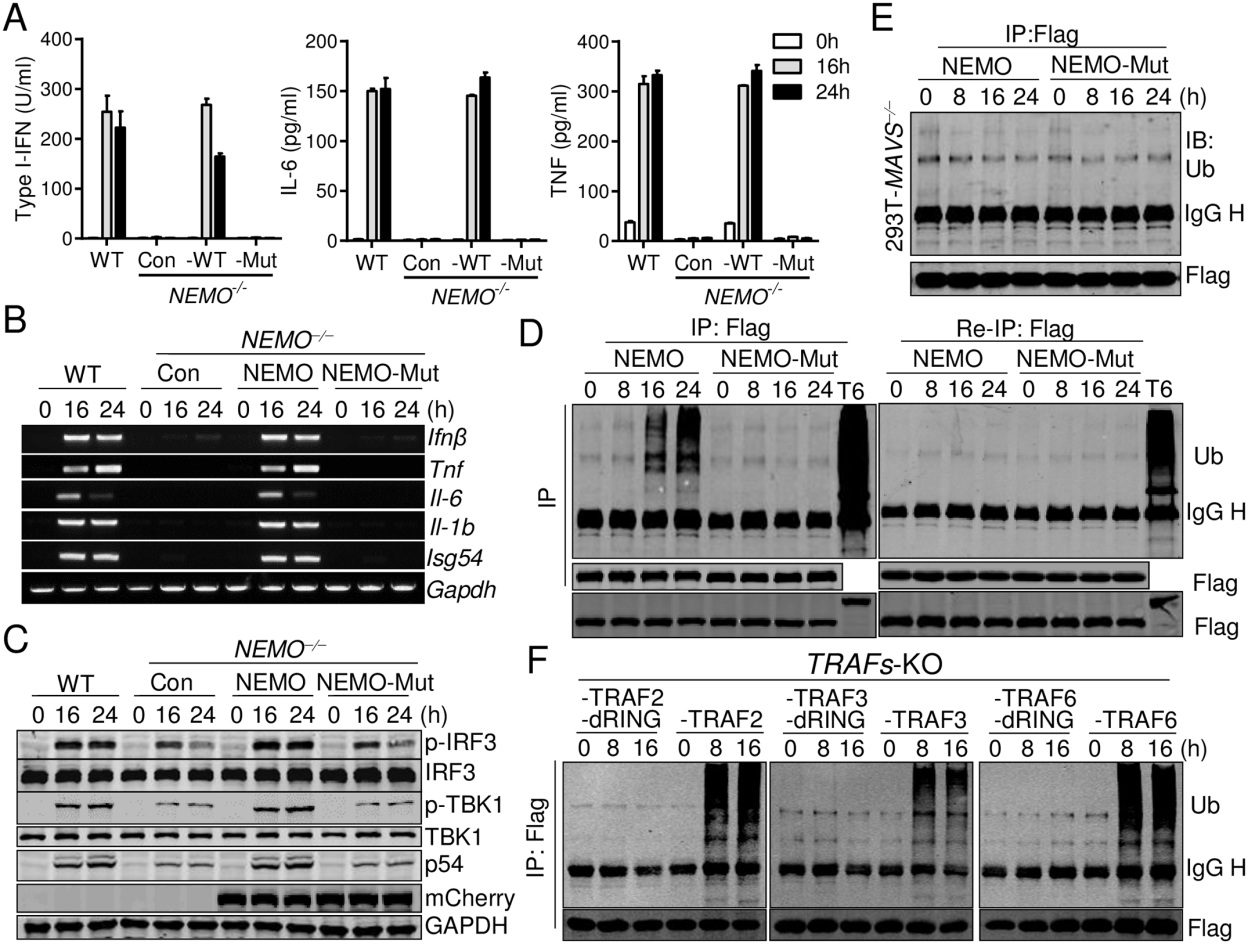
NEMO is activated through the binding of ubiquitin chains synthetized by TRAFs. (A-C) WT, *NEMO*^*−/−*^ and *NEMO*^*−/−*^ THP-1 cells reconstituted with mCherry-tagged NEMO or mCherry-tagged NEMO-Mut (Y^308^S/D^311^N/H^413^A/C^417^A) were infected with SeV for the indicated times. Type I-IFNs, IL-6 and TNF were determined by bioassay or ELISA (A). The indicated gene induction was analyzed by RT-PCR (B). Cell lysates were analyzed by Western blot to detect the phosphorylation and expression of the indicated proteins (C). (D) 293T cells stably expressing Flag-tagged NEMO or Flag-tagged NEMO-Mut were infected with SeV for the indicated times and 293T cells were transiently transfected with Flag-TRAF6 for 24 h. Cell lysates were immunoprecipitated with the anti-Flag antibody. Half of the precipitates were subjected to a second-round immunoprecipitation. The precipitates were analyzed by Western blot with the anti-ubiquitin and ant-Flag antibodies (Upper panels). The whole cell lysates (WCL) were analyzed with the anti-Flag antibody to detect the expression of Flag-tagged NEMO and TRAF6 (Bottom). (E) to (F) The same experiment was performed as in (D) except that 293T *MAVS*^*−/−*^ cells stably expressing Flag-NEMO or Flag-NEMO-Mut (E) or TRAFs-deficient cells reconstituted with TRAF2, 3, or 6 or TRAF2/3/6-dR stably expressing Flag-tagged NEMO (F) were used. The precipitates were analyzed by Western blot with the anti-ubiquitin (Top) and ant-Flag (Bottom) antibodies. Data from (A) represent mean ± SD. Similar results were obtained in 3 independent experiments.

Our results thus far suggested that NEMO activation depended on TRAFs’ E3 ligase activity. Since previous work showed that the NEMO mutant in which 13 conserved lysine residues were substituted with arginine still supported IRF3 activation in *Nemo*^*−/−*^ MEFs [27], we tested whether ubiquitin binding of NEMO was important for MAVS-mediated signaling. NEMO was reported to bind K63 or linear ubiquitin chains through two ubiquitin binding domains: a NEMO–ubiquitin binding domain and a C-terminal zinc finger domain [46]. Two critical amino acids in each domain were replaced to generate the NEMO mutant that had no ubiquitin binding activities (Y^308^S/D^311^N/H^413^A/C^417^A) [27]. NEMO-WT or NEMO-Mut was stably expressed in *NEMO*^*−/−*^ THP-1 cells and tested for SeV responses. Only NEMO-WT but not NEMO-Mut restored the response of *NEMO*^*−/−*^ cells to SeV, as type I-IFN, IL-6 and TNF induction was only detected in NEMO-WT-reconstituted cells (Fig 6A), which was confirmed at the mRNA level (Fig 6B). Consistent with these findings, TBK1 and IRF3 phosphorylation was fully restored with the reconstituted NEMO-WT, but not with the mutant (Fig 6C), suggesting that ubiquitin chains produced by TRAFs bound to and activated NEMO.

Next, we assessed whether NEMO indeed interacted with ubiquitin chains. NEMO was immunoprecipitated from SeV-infected 293T cells stably expressing Flag-tagged NEMO. Half of the immunoprecipitated NEMO was denatured to perform the second immunoprecipitation. The precipitates were then analyzed by an anti-ubiquitin antibody. We found that SeV infection induced a vigorous ubiquitin chain binding on NEMO-WT, but not NEMO-Mut (Fig 6D, left). However, this ubiquitin binding was removed by the denaturation process in the second-step immunoprecipitation, whereas TRAF6-mediated polyubiquitination of itself was still detected (Fig 6D, right), suggesting that NEMO interacted with the ubiquitin chains. In MAVS-deficient cells, however, neither NEMO-WT nor NEMO-Mut was found to bind ubiquitin chains after viral infection (Fig 6E), indicating that MAVS activation initiated ubiquitin chain synthesis and subsequent NEMO binding. Critically, such ubiquitin chain binding was only observed in TRAFs-deficient cells reconstituted with the wildtype TRAF, but not with TRAF-dRing mutants (Fig 6F), confirming that TRAFs’ E3 ligase was responsible for the ubiquitin chain synthesis. These results collectively suggested that NEMO was activated by the binding of ubiquitin chains synthetized by TRAFs.

### IKKα/β are critical for NEMO-mediated TBK1/IKKε activation in the RLR-MAVS pathway

**Fig 7:**
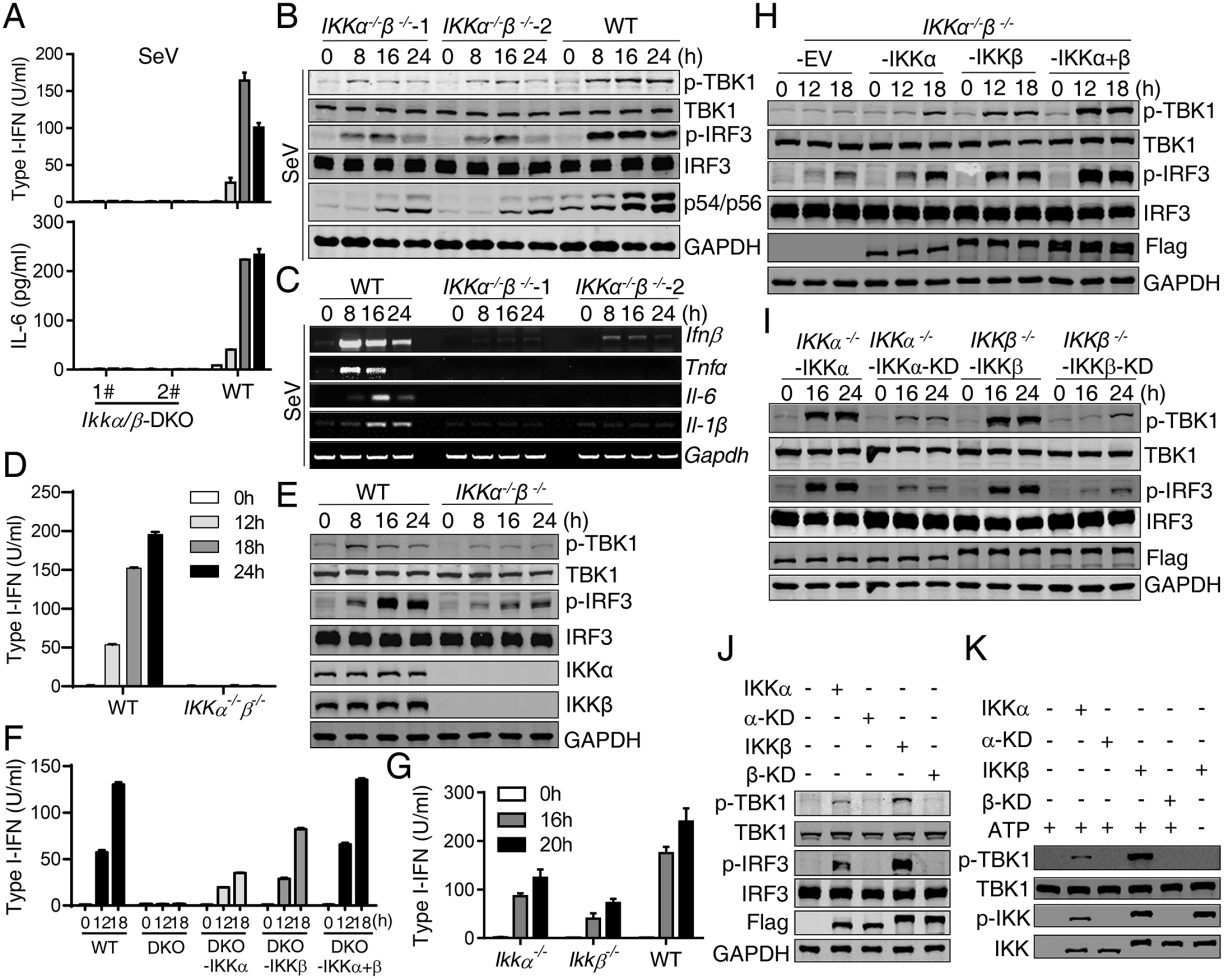
IKKα/β are critical for MAVS-mediated TBK1/IKKε activation. (A) to (C) WT and *IKKα*^*−/−*^*IKKβ*^*−/−*^ HeLa cells were infected with SeV for the indicated times. Type I-IFN and IL-6 production was determined by bioassay or ELISA respectively (A). Cell lysates were analyzed by Western blot to detect the phosphorylation and expression of the indicated proteins (B). The indicated gene induction was analyzed by RT-PCR (C). (D) to (E) WT and *IKKα*^*−/−*^*IKKβ*^*−/−*^THP1 cells were infected with SeV for the indicated times. Type I-IFN production was determined by bioassay (D). Cell lysates were analyzed by Western blot to detect the phosphorylation and expression of the indicated proteins (E). (F) WT, *IKKα*^*−/−*^*IKKβ*^*−/−*^ and *IKKα*^*−/−*^*IKKβ*^*−/−*^ HeLa cells reconstituted with IKKα, IKKβ or IKKα/β together were infected with SeV for the indicated times. Type I-IFN production was determined by bioassay. (G) WT, *IKKα*^*−/−*^ and *IKKβ*^*−/−*^ HeLa cells were infected with SeV for the indicated times. Type I-IFN production was determined by bioassay. (H) *IKKα*^*−/−*^*IKKβ*^*−/−*^ HeLa cells reconstituted with empty vector (EV), IKKα, IKKβ or IKKα/β together were infected with SeV for the indicated times. Cell lysates were analyzed by Western blot to detect the phosphorylation and expression of the indicated proteins. (I) *IKKα*^*−/−*^ HeLa cells reconstituted with IKKα or IKKα-KD (IKKα-D^144^A) and *IKKβ*^*−/−*^ HeLa cells reconstituted with IKKβ or IKKβ-KD (IKK*β*-D^145^A) were infected with SeV for the indicated times. Cell lysates were analyzed by Western blot to detect the phosphorylation and expression of the indicated proteins. (J) 293T cells were transfected with the indicated plasmids for 24 h. Cell lysates were analyzed by Western blot to detect the phosphorylation of the indicated proteins. (K) Flag-tagged IKKα, IKKα-KD, IKKβ or IKKβ-KD protein, stably expressed in *IKKα*^*−/−*^*IKKβ*^*−/−*^ HeLa cells, was immunoprecipitated after cells were infected with SeV for 18 h, and incubated with recombinant TBK1 at room temperate for 30 min, the phosphorylation of the indicated proteins was detected by Western blot. Data from (A), (D), (F) and (G) represent mean ± SD. Similar results were obtained in 3 independent experiments. See also S7 Fig.

Since NEMO was required for the full activation of TBK1/IKKε, we explored how NEMO activated TBK1/IKKε by asking whether NEMO-mediated IKKα/β activation was involved in the TBK1/IKKε activation. To address this question, IKKα and IKKβ double-deficient HeLa cells were generated (S7A Fig). As expected, this *IKKα*^*−/−*^*β*^*−/−*^ cell demonstrated severely diminished responses to SeV infection (Fig 7A). However, similar to that observed in NEMO-deficient cells, the phosphorylation of TBK1, IRF3 and the induction of ISGs were all significantly reduced in this double deficient cell (Fig 7B), suggesting that IKKα/β were indeed required for TBK1/IKKε activation. Similar results were obtained in *IKKα*^*−/−*^*β*^*−/−*^ THP1 cells (Fig 7D and 7E). These results strongly suggested that NEMO operated through IKKα and/or IKKβ to promote TBK1/IKKε activation in the RLR-MAVS pathway.

Next, we reconstituted the double-deficient cells with IKKα, IKKβ or IKKα/β to determine the importance of each kinase. We found that IKKα and IKKβ are both required in the RLR-MAVS pathway, as re-expression of either IKKα or IKKβ only partially restored SeV-induced type I-IFN production (Fig 7F), in which the kinase activity was important (Fig 7I). To further determine each IKK kinase’s role, HeLa cells deficiency of either IKKα or IKKβ were made (S7B and S7C Fig) and infected with SeV. Deficiency of either IKKα or IKKβ showed partial impairment of type I-IFN induction in response to SeV infection (Fig 7G).

Our results indicated that NEMO-IKKα/β affected MAVS signaling by activating NF-κB and promoting TBK1/IKKε activation. Previous work demonstrated that IKKβ phosphorylated TBK1 and IKKε at Ser^172^ when IKKβ was overexpressed or upon TLR activation [47, 48]. When overexpressed, we confirmed that both IKKα and IKKβ but not IKKα-KD or IKKβ-KD caused TBK1 phosphorylation (Fig 7J) in 293T cells, in which IKKβ had a stronger impact. Consistently, co-expression of TBK1 with IKKβ, but not with IKKβ-KD, resulted in highly elevated type I-IFN production and TBK1/IRF3 phosphorylation (S7D Fig). When IKKα was co-expressed with TBK1, it also caused enhanced TBK1/IRF3 phosphorylation and IFN production, but to a reduced extent. Importantly, recombinant TBK1 purified from *E*. *coli* was phosphorylated at Ser^172^ by the immunoprecipitated IKKα or IKKβ from SeV-infected cells *in vitro* (Fig 7K). Based on these results, we concluded that IKKα/β directly phosphorylated and thus promoted TBK1 and IKKε activation during SeV infection.

## Discussion

Although it is well known that MAVS activates the transcription factor IRF3 through TBK1/IKKε after RNA virus infection, the mechanism by which TBK1/IKKε are recruited and activated remained unclear. Using TANK/SINTBAD/NAP1 triple deficient cells, we unambiguously demonstrated that none of these TBK1-binding proteins is required for RLR-MAVS activation. Instead, we presented several lines of evidence to show that MAVS activated TBK1/IKKε through TRAF proteins. First, NEMO was not required for MAVS-TBK1/IKKε interaction. Second, combined deletion of TRAFs completely abolished MAVS-TBK1/IKKε interaction and the subsequent activation of TBK1/IKKε. Third, TRAFs activation of TBK1/IKKε was independent of its E3 ligase activity in the absence of NEMO. Finally, TRAF proteins interacted with TBK1/IKKε directly and the minimum element for interaction was also the minimum part to activate TBK1/IKKε *ex vivo*. Based on these results, we propose a working model in which MAVS recruits and activates TBK1/IKKε via pre-associated TRAFs-TBK1/IKKε complex upon RNA virus infection (S7E Fig). Importantly, since TBK1-IRF3 activation and type I-IFN production were detected in NEMO-deficient 293T and MEF cells, in which NF-κB activation was completely lost, our data also indicate that although NF-κB activation is important for the full induction of type I-IFNs, a minimal amount of IFNs is still produced in the absence of NF-κB activation.

Previous structural studies showed that TBK1 is activated through trans-autophosphorylation [40]. Adaptor proteins are required to bring TBK1 dimers within close proximity and appropriate orientation for its activation. We found that TBK1/IKKε constitutively associated with TRAF proteins in resting cells. Upon infection, the TRAFs-TBK1/IKKε complex was recruited to MAVS, likely bringing TBK1/IKKε close enough to cause the trans-autophosphorylation. Further structural studies on the TRAFs-TBK1/IKKε complex will help to verify how TBK1/IKKε are autophosphorylated. Our finding that TBK1/IKKε were pre-associated with TRAFs is consistent with previous data showing that TBK1 constitutively formed a complex with TRAF2, from which TBK1 was identified and named as T2K [38]. Importantly, the bacterially expressed TBK1 and TRAFs displayed strong and direct interactions, even between TBK1 and TRAF5, which was hardly detected in cells. Given the fact that type I-IFNs are among the first produced cytokines to initiate a globally cellular antiviral state, the pre-associated TRAF-TBK1 complex may facilitate the induction of IFNs at the very beginning of viral infection. Indeed, this kind of pre-associated protein complex is not unusual, with the IKK-complex being a good example [49]. In addition, TAK1-TAB1-TAB2 was found to pre-associate together in resting cells, and was recruited by IRAK-TRAF6 upon TLR/IL-1R activation [50].

We discovered that NEMO is absolutely required for MAVS-induced IKKα/β activation and is also important for the full activation of TBK1/IKKε. Previous work demonstrated that the ubiquitin binding-defective NEMO failed to restore TBK1 and IRF3 phosphorylation *in vitro* [26]. Recent studies also demonstrate that NEMO plays a critical role in IRF3/7 activation in the RLR-MAVS pathway [31]. These findings indicate a role of NEMO in the activation of TBK1/IKKε in RNA virus infected cells. However, since NEMO binds neither to TBK1 nor to IKKε [31, 51], how NEMO manages to activate TBK1/IKKε remains elusive. In this study, we found that NEMO-activated IKKα/β coupled NEMO to TBK1/IKKε activation. IKKα/β overexpression or immunoprecipitated IKKα/β from SeV stimulated cells caused a strong TBK1 phosphorylation at Ser^172^, a critical phosphorylation site for this kinase to be activated. This result agrees with previous work [47, 48]. We thus propose that IKKα/β is required for the activation of TBK1/IKKε by directly phosphorylating these kinases, thus enhancing the activation of IRF3.

Our results demonstrated that different TRAFs functioned downstream of MAVS to recruit and activate TBK1/IKKε. The physiological situation could be more complicated. Since the expression patterns of TRAFs are quite different in various tissues and cells [52–55], and the extent of RLR activation was not the same in each TRAF-reconstituted cell, the redundancy may not be physiologically implemented. The use of complex downstream adaptor proteins will confer cells elaborate ways to regulate the strength or magnitude of innate immune activation among different cells and tissues after viral invasion, which certainly would be beneficial to the host. In addition, different TRAFs may form hetero-oligomers to activate TBK1 and IKKε differentially, in this way further fine-tuning virus infection triggered innate immune responses.

## Materials and methods

### Reagents

Thrombin (T4648), all chemicals were purchased from Sigma-Aldrich Corp. (St. Louis, MO), unless otherwise indicated. Transfection was done with Lipofectamine 2000 (Invitrogen). Ni-NTA beads were purchased from Clontech (635660). Anti-MAVS (Cell signaling, #3993), anti-IRF3 (Santa Cruz, sc-9082), anti-Flag (Sigma, F3165), anti-HA (Sigma, H9658), anti-GAPDH (Santa Cruz, sc-25778), anti-TBK1 (Santa Cruz, sc-52957; Cell signaling, #3504), anti-IKKε (Cell signaling, #2690S), anti-NEMO (Cell signaling, #2686S), anti-His (cw00c28), anti-IκBα (Cell signaling, #4814), anti-p-IRF3 (Epitomics, 2562–1), anti-p-TBK1 (Abcam, ab109272), anti-p-IκBα (Cell signaling, #2859), anti-p-IKKα/β (Cell signaling, #2078), anti-IKKα (Cell signaling, #2682), anti-IKKβ (Cell signaling, #2370), anti-NAP1 (Proteintech, 15042-1-AP), anti-TANK (Bioworld, BS2231), anti-SINTBAD (Cell signaling, #8605) antibodies were purchased as indicated. Antisera against viperion and p54/56 were generated by immunizing mice with the full length recombinant protein produced in *E*. *coli*, at Beijing Biotop Biotechnology, China.

### Cells and viruses

HEK293T and its derived knockout cells, HeLa and its derived knockout cells, 2FTGH-ISRE, wild-type and *Nemo*^−/−^ MEFs cells were cultured in DMEM (GIBCO), supplemented with 10% FBS (GIBCO), 5 mg/ml penicillin (GIBCO) and 10 mg/ml streptomycin (GIBCO). THP1 and its derived knockout cells were cultured in RPMI-1640 (Gibco) media supplemented with 10% FBS (GIBCO). Sendai virus was obtained from Congyi Zheng, Wuhan University, China.

### Plasmids

cDNA coding human TRAF2, 3, 5, or 6 was amplified from 293T, U937 or B cells, and cloned into pSin-EF2-IRES-Puro (Addgene) to generate Flag-tagged proteins. Deleted, truncated and point mutants were generated by the QuickChange site-directed mutagenesis kits with pfu-Ultra as the polymerase (Stratagene, La Jolla, CA) and the construct coding the wild-type protein as the template. Other expressing plasmids were described previously [56–58]. pU6-sgRNA and pCDNA3.1-CAS9 were used as described [59]. Both sgRNA and Cas9 were cloned into pSin-EF2-IRES-Puro (Addgene) for lentivirus Packaging. Each construct was confirmed by sequencing.

### Co-immunoprecipitation, Immunoblot analysis, RT-PCR, Type I-IFN bioassay, Luciferase reporter assay

These experiments were performed as previously described [56–58].

### Enzyme-linked immunosorbent assay (ELISA)

Cell culture supernatants, tissue homogenates and sera were collected, levels of IL-6 (eBioscience, #88–7066) or TNFα (eBioscience, #88–7346) were measured according to manufacturer’ s instructions.

### Recombinant protein purification and pull-down experiments

The expression and purification of recombinant proteins were performed as previously described [60]. TRAF2/3/5/6 were cloned into the prokaryotic expression vector PET28a and purified with Ni-NTA beads. TBK1 with the C-terminal Flag tag was cloned into PET28a, after purified with Ni-NTA beads, the N-terminal His tag was cleaved by Thrombin. Ni-NTA beads was used again to remove the proteins that didn’t cleave by Thrombin. For the pull-down experiments, 10 μg TBK1-Flag and 50 μg His-TRAF2/3/5/6/2C were diluted to 1 ml with PBS and incubated with 20 μl Ni-NTA beads in 4°C for 2 h, then the Ni-NTA beads were collected and washed with PBS containing 30 mM imidazole for three times, then the proteins were eluted with 50 μl 1xSDS buffer and boiled 10 mins in 100°C. 5 μl of the eluates was used for immunoblot analysis.

### Knockout cell generation and restoration

All the gene-specific deficient cell lines in HEK293T, HeLa and THP1 were generated with CRISPR-Cas9-mediated gene targeting. 293T cells were co-transfected with pSin-sgRNA and pCDNA3.1-Cas9 for 24 h and selected by puromycin (2 μg/ml), cells resistant to puromycin were sorted by FACS into 96-well plate and single cell clones were picked and identified. The procedures of generating knockout cell lines in HeLa or THP1 were same to that in 293T except the sgRNAs were transduced by lentivirus. The target sequences of sgRNAs are shown in the table. To generate transgenic stable expression cell lines, cells were infected with lentivirus of the transgene and selected by puromycin (2 μg/ml). The lentivirus was produced by co-transfection of pSin-EF2-IRES-Puro vectors with two viral packaging plasmids pMD2.G (Addgene) and psPAX2 (Addgene) into HEK293T cells by standard calcium phosphate precipitation method.

### Statistical analysis

We performed statistical analysis by using an unpaired Student’s *t*-test for all studies unless otherwise indicated.

## Supporting information

S1 Fig TBK1-binding proteins TANK/NAP1/SINTBAD are not required for RLR-MAVS pathway. (A) to (D) The deletion of TANK/NAP1/SINTBAD in *TANK*^*−/−*^*NAP1*^*−/−*^*SINTBAD*^*−/−*^ 293T cells was detected with Genotyping (A, B, C) and Western blot analysis (D). (E) to (G) WT and *TANK*^*−/−*^*NAP1*^*−/−*^*SINTBAD*^*−/−*^ 293T cells were infected with SeV for the indicated times. Cells were analyzed by Western blot to detect the phosphorylation and expression of the indicated proteins (E), or RT-PCR analysis of the indicated gene induction (F), bioassay analysis of type I-IFN production (G). Data from (G) represent mean ± SD. Similar results were obtained in 3 independent experiments.
(PDF)

S2 Fig TRAFs are absolutely required for MAVS-mediated signaling. Related to Fig 1. (A) to (D) Genotyping of *TRAF2*^*−/−*^ (A), *TRAF3*^*−/−*^ (B), *TRAF5*^*−/−*^ (C) and *TRAF6*^*−/−*^ (D) 293T cells. (E) WT and *TRAF6*^*−/−*^ 293T cells were infected with SeV for the indicated times, cells were analyzed by Western blot to detect the phosphorylation and expression of the indicated proteins. (F) WT and *Traf2*^*−/−*^, *Traf3*^*−/−*^ and *Traf6*^*−/−*^ MEF cells were infected with SeV for the indicated times. Supernatants were analyzed by bioassay to detect type I-IFN production (upper). Cells were analyzed by Western blot to detect the phosphorylation and expression of the indicated proteins (lower). (G) Expression of the reconstituted proteins in TRAFs-deficient 293T cells reconstituted with TRAF2, 3, or 6 and the endogenous proteins was determined by Western blot (left). Cells were infected with SeV for the indicated times, type I-IFN production was analyzed with bioassay (right). (H) Genotyping of TRAFs-deficient 293T cells. Data from (F), (G) represent mean ± SD. Similar results were obtained in 3 independent experiments.
(PDF)

S3 Fig TBK1/IKKe are recruited to MAVS via the pre-associated TRAFs-TBK1/IKKe. Related to Fig 2. (A) 293T cells were transfected with Flag-tagged TRAFs and full length IKKε or IKKε truncations illustrated in the upper panel for 24 h. Cell lysates were immunoprecipitated with the anti-Flag antibody. The precipitates and whole cell lysates (WCL) were analyzed by Western blot with the indicated antibodies. Truncations 1 to 4 indicate IKKε and IKKε lacking amino acids 304–382, 609–648 and 649–716. (B) *MAVS*^−/−^ THP1 cells reconstituted with MAVS-WT were infected with SeV for the indicated times, cell lysates were immunoprecipitated with the anti-HA or anti-TANK antibody. The precipitates and whole cell lysates (WCL) were analyzed by Western blot with the indicated antibodies. Data are representatives of three experiments.
(PDF)

S4 Fig MAVS activates TBK1/IKKε in both NEMO-dependent and independent manners. Related to Fig 3. (A) to (C) Genotyping of *NEMO*^*−/−*^ 293T cells (A), *NEMO*^*-/-*^ HeLa and THP1 cells (B), *MAVS*^*−/−*^ 293T cells (C). (D) WT, *MAVS*^−/−^ and *NEMO*^−/−^ 293T cells were infected with SeV for the indicated times. Indicated gene induction was analyzed by RT-PCR. (E) Genotyping of WT MEFs or MEFs deficient of *Nemo*. Upper: schematic diagram of the mouse *Nemo* locus and primers for genotyping. Exons 1 and 2 are indicated by solid boxes. The translation start site, selection markers, PCR screening primers (P1, P2, P3 and P4), and restriction enzyme sites are shown. B, BamHI; Bg, BglII; 47III, Eco47III; S, SalI. Lower: the PCR products were examined by agarose gel electrophoresis. Primers (P1, P2, P3 and P4) are shown in Supplementary materials. (F) WT and *Nemo*^−/−^ MEF cells were infected with SeV for the indicated times. Indicated gene induction was analyzed by RT-PCR.
(PDF)

S5 Fig TRAFs’ E3 ligase activity is required for TBK1/IKKε activation via NEMO. Related to Fig 4. (A) Genotyping of *TRAFs-NEMO*-deficient 293T cells at the locus of *NEMO*. (B) to (C) Expression of the reconstituted proteins as indicated in cells used in Fig 4A (B) and Fig 6C (C) and the endogenous proteins was determined by Western blot.
(PDF)

S6 Fig TRAFs’ coiled-coil domain is important for interaction and activation of TBK1/IKKε. Related to Fig 5. (A) to (C) 293T cells were transfected with Flag-tagged TRAFs or the indicated truncations and HA-tagged TBK1(A), HA-tagged IKKε (B) or HA-tagged MAVS (C) for 24 h. Cell lysates were immunoprecipitated with the anti-Flag antibody. The precipitates and whole cell lysates (WCL) were analyzed by Western blot with the indicated antibodies.
(PDF)

S7 Fig IKKα/β are critical for MAVS-mediated TBK1/IKKε activation. Related to Fig 7. (A) Genotyping of *IKKα*^*-/-*^*IKKβ* ^*-/-*^ HeLa cells. (B) to (C) The deletion of IKKα or IKKβ in *IKKα*^*−/−*^ or *IKKβ*^*−/−*^ HeLa cells was detected with Genotyping (B) and Western blot analysis (C). (D) 293T cells were transfected with P651–Luc reporter (50 ng) and the indicated plasmids (IKKα 300 ng, IKKβ 300 ng, IKKβ-KD 300 ng, TBK1 50 ng). Luciferase assay was performed after 24 h. (E) Working model. Upon binding of dsRNA, RIG-I undergoes conformational changes and releases the N-terminal tandem CARD domains. The exposed CARDs of RIG-I activate MAVS by inducing MAVS polymerization through CARD-CARD interaction. MAVS polymers then recruit the pre-associated TRAFs-TBK1/IKKε complex, leading to TBK1/IKKε activation by trans-autophosphorylation. Meanwhile, oligomeric TRAFs synthesize K63-linked polyubiquitin chains to activate the NEMO-IKKa/β complex, which further activate TBK1/IKKε. Fully activated TBK1/IKKε and IKKa/β phosphorylate and activate transcriptional factors IRF3/7 and NF-κB respectively, which translocate into the nucleus to induce the production of various cytokines including type I-IFNs.
(PDF)

S1 Text Extended experimental procedures. (DOC)
