# Supplementary figures and images for: MAVS activates TBK1 and IKKε through TRAFs in NEMO dependent and independent manner
Source: PLoS Pathog. 2017 Nov 10;13(11):e1006720. doi: 10.1371/journal.ppat.1006720 (PMC5699845; doi:10.1371/journal.ppat.1006720)

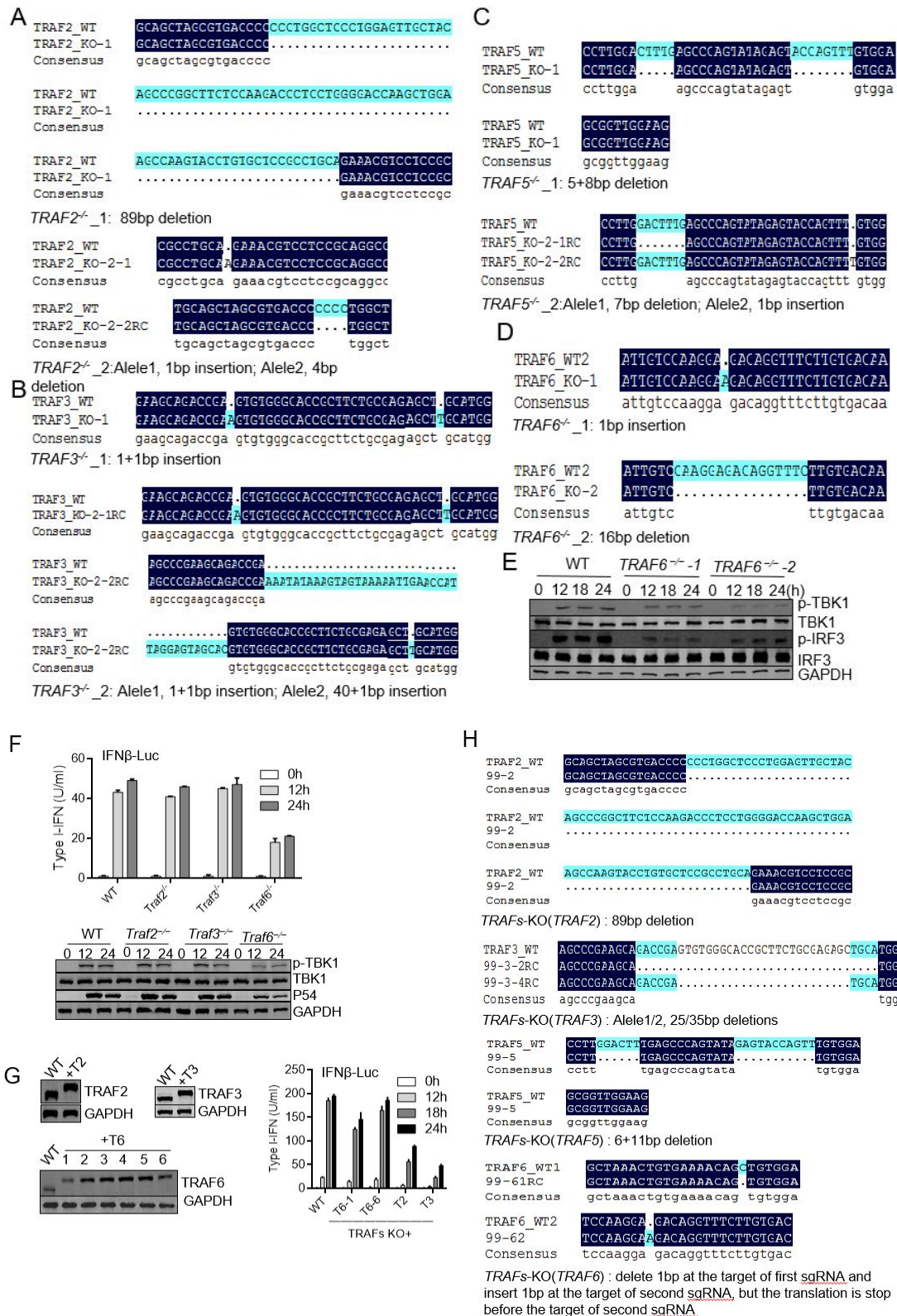

**Fig S2. TRAFs are absolutely required for MAVS-mediated signaling. Related to Fig 1**

Supplement: S2 Fig — (A) to (D) Genotyping of TRAF2−/− (A), TRAF3−/− (B), TRAF5−/− (C) and TRAF6−/− (D) 293T cells. (E) WT and TRAF6−/− 293T cells were infected with SeV for the indicated times, cells were analyzed by Western blot to detect the phosphorylation and expression of the indicated proteins. (F) WT and Traf2−/−, Traf3−/− and Traf6−/− MEF cells were infected with SeV for the indicated times. Supernatants were analyzed by bioassay to detect type I-IFN production (upper). Cells were analyzed by Western blot to detect the phosphorylation and expression of the indicated proteins (lower). (G) Expression of the reconstituted proteins in TRAFs-deficient 293T cells reconstituted with TRAF2, 3, or 6 and the endogenous proteins was determined by Western blot (left). Cells were infected with SeV for the indicated times, type I-IFN production was analyzed with bioassay (right). (H) Genotyping of TRAFs-deficient 293T cells. Data from (F), (G) represent mean ± SD. Similar results were obtained in 3 independent experiments. (PDF) [file ppat.1006720.s002.pdf]

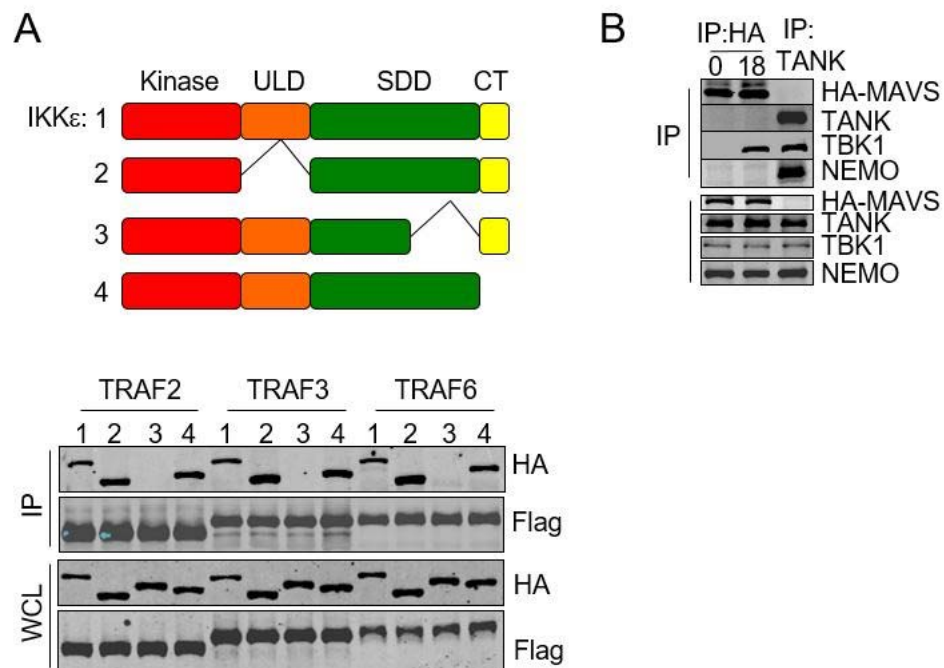

**Fig S3. TBK1/IKKε are recruited to MAVS via the pre-associated TRAFs-TBK1/IKKε.**

Related to Fig 2

Supplement: S3 Fig — (A) 293T cells were transfected with Flag-tagged TRAFs and full length IKKε or IKKε truncations illustrated in the upper panel for 24 h. Cell lysates were immunoprecipitated with the anti-Flag antibody. The precipitates and whole cell lysates (WCL) were analyzed by Western blot with the indicated antibodies. Truncations 1 to 4 indicate IKKε and IKKε lacking amino acids 304–382, 609–648 and 649–716. (B) MAVS−/− THP1 cells reconstituted with MAVS-WT were infected with SeV for the indicated times, cell lysates were immunoprecipitated with the anti-HA or anti-TANK antibody. The precipitates and whole cell lysates (WCL) were analyzed by Western blot with the indicated antibodies. Data are representatives of three experiments. (PDF) [file ppat.1006720.s003.pdf]

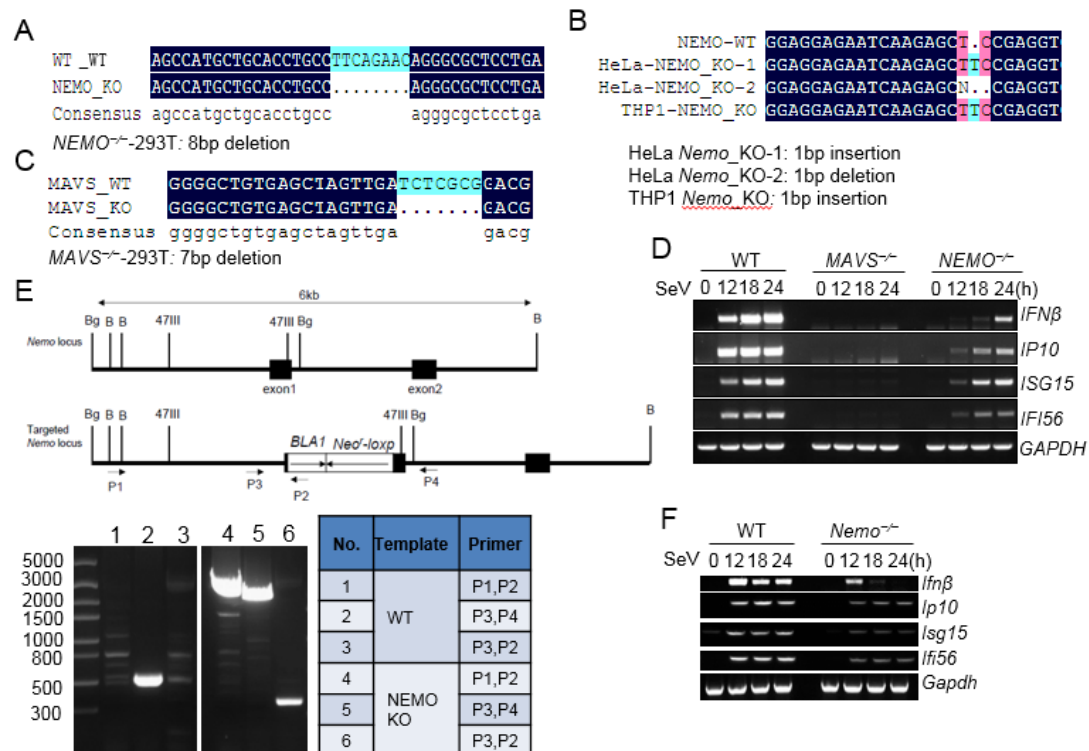

**Fig S4. MAVS activates TBK1/IKKε in both NEMO-dependent and independent manners.**

Related to Fig 3

Supplement: S4 Fig — (A) to (C) Genotyping of NEMO−/− 293T cells (A), NEMO-/- HeLa and THP1 cells (B), MAVS−/− 293T cells (C). (D) WT, MAVS−/− and NEMO−/− 293T cells were infected with SeV for the indicated times. Indicated gene induction was analyzed by RT-PCR. (E) Genotyping of WT MEFs or MEFs deficient of Nemo. Upper: schematic diagram of the mouse Nemo locus and primers for genotyping. Exons 1 and 2 are indicated by solid boxes. The translation start site, selection markers, PCR screening primers (P1, P2, P3 and P4), and restriction enzyme sites are shown. B, BamHI; Bg, BglII; 47III, Eco47III; S, SalI. Lower: the PCR products were examined by agarose gel electrophoresis. Primers (P1, P2, P3 and P4) are shown in Supplementary materials. (F) WT and Nemo−/− MEF cells were infected with SeV for the indicated times. Indicated gene induction was analyzed by RT-PCR. (PDF) [file ppat.1006720.s004.pdf]

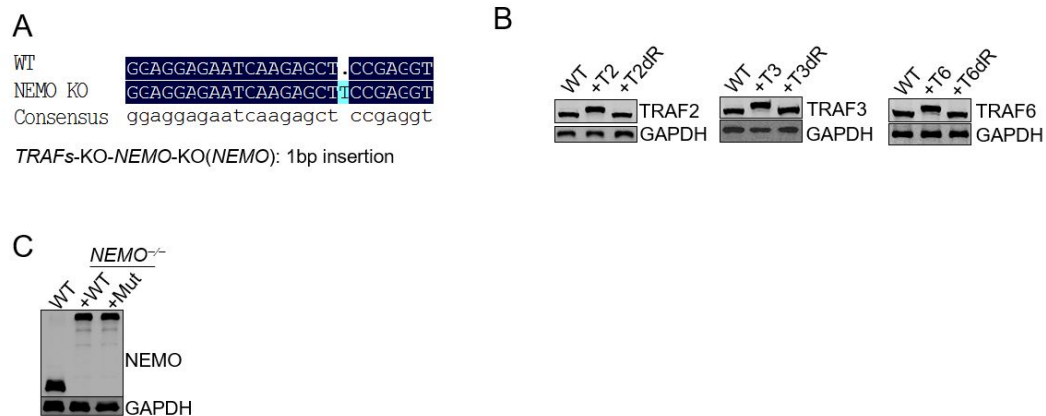

**Fig S5. TRAFs' E3 ligase activity is required for TBK1/IKK $\epsilon$  activation via NEMO.**

**Related to Fig 4**

Supplement: S5 Fig — (A) Genotyping of TRAFs-NEMO-deficient 293T cells at the locus of NEMO. (B) to (C) Expression of the reconstituted proteins as indicated in cells used in Fig 4A (B) and Fig 6C (C) and the endogenous proteins was determined by Western blot. (PDF) [file ppat.1006720.s005.pdf]

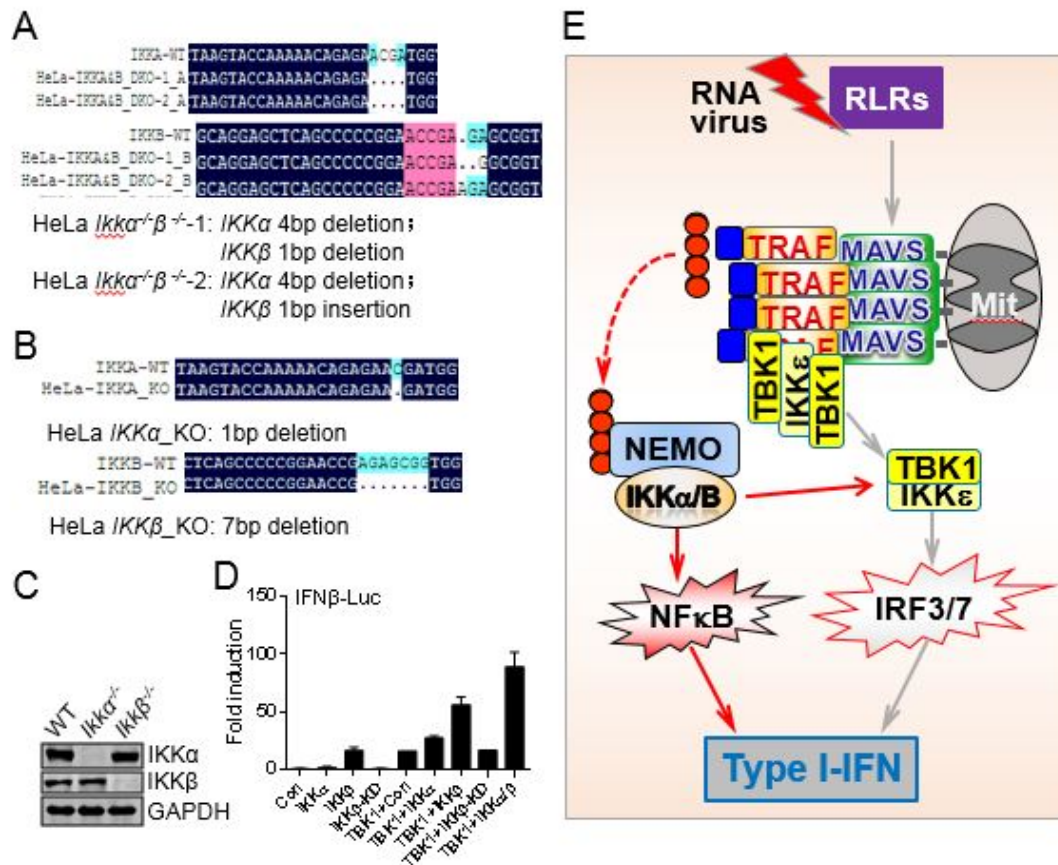

Fig S7. IKK $\alpha$ / $\beta$  are critical for MAVS-mediated TBK1/IKK $\epsilon$  activation. Related to Fig 7

Supplement: S7 Fig — (A) Genotyping of IKKα-/-IKKβ -/- HeLa cells. (B) to (C) The deletion of IKKα or IKKβ in IKKα−/− or IKKβ−/− HeLa cells was detected with Genotyping (B) and Western blot analysis (C). (D) 293T cells were transfected with P651–Luc reporter (50 ng) and the indicated plasmids (IKKα 300 ng, IKKβ 300 ng, IKKβ-KD 300 ng, TBK1 50 ng). Luciferase assay was performed after 24 h. (E) Working model. Upon binding of dsRNA, RIG-I undergoes conformational changes and releases the N-terminal tandem CARD domains. The exposed CARDs of RIG-I activate MAVS by inducing MAVS polymerization through CARD-CARD interaction. MAVS polymers then recruit the pre-associated TRAFs-TBK1/IKKε complex, leading to TBK1/IKKε activation by trans-autophosphorylation. Meanwhile, oligomeric TRAFs synthesize K63-linked polyubiquitin chains to activate the NEMO-IKKa/β complex, which further activate TBK1/IKKε. Fully activated TBK1/IKKε and IKKa/β phosphorylate and activate transcriptional factors IRF3/7 and NF-κB respectively, which translocate into the nucleus to induce the production of various cytokines including type I-IFNs. (PDF) [file ppat.1006720.s007.pdf]
