# Supplementary material for: MAVS activates TBK1 and IKKε through TRAFs in NEMO dependent and independent manner
Source: PLoS Pathog. 2017 Nov 10;13(11):e1006720. doi: 10.1371/journal.ppat.1006720 (PMC5699845; doi:10.1371/journal.ppat.1006720)
Supplement: S6 Fig — (A) to (C) 293T cells were transfected with Flag-tagged TRAFs or the indicated truncations and HA-tagged TBK1(A), HA-tagged IKKε (B) or HA-tagged MAVS (C) for 24 h. Cell lysates were immunoprecipitated with the anti-Flag antibody. The precipitates and whole cell lysates (WCL) were analyzed by Western blot with the indicated antibodies. (PDF) [file ppat.1006720.s006.pdf]

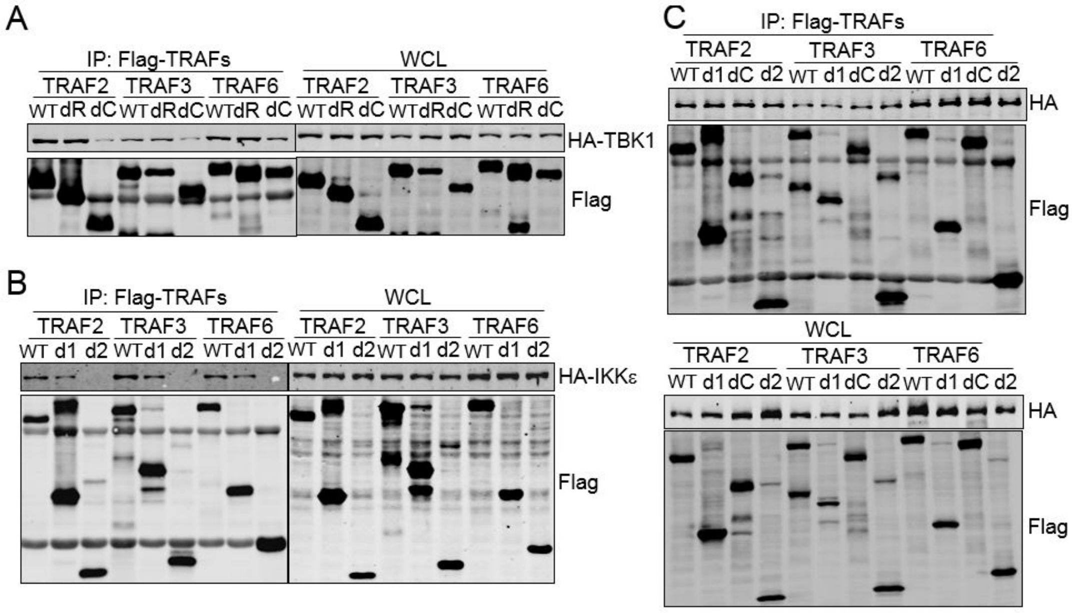

Fig S6. TRAFs' coiled-coil domain is important for interaction and activation of TBK1/IKKε.

Related to Fig 5
