# Supplementary material for: MAVS activates TBK1 and IKKε through TRAFs in NEMO dependent and independent manner
Source: PLoS Pathog. 2017 Nov 10;13(11):e1006720. doi: 10.1371/journal.ppat.1006720 (PMC5699845; doi:10.1371/journal.ppat.1006720)
Supplement: S1 Text — (DOC) [file ppat.1006720.s008.doc]

**Extended experimental procedures**

**Coimmunoprecipitation** **and immunoblot analysis**

Protein extracts were prepared by standard techniques and were performed by co-immunoprecipitation (Co-IP) and immunoblot as described [1]. For endogenous Co-IP experiments, cells (2×10^7^) were lysed in 1 ml of lysis buffer, the subsequent procedures were carried out as described [1].

**RT-PCR and Q-PCR**

Total RNA was extracted with Trizol reagent (Transgen) and reversed-transcribed with Reverse Transcription System (Promega). Type I-interferon, ISGs induction was analyzed by RT-PCR for 28-30 cycles at 94°C for 30s, 58°C for 30s and 72°C for 30s. The primers are shown in the following table. Reverse transcription products of different samples were amplified by ABI 7300 Detection System (Applied Biosystems) using the SYBR Green PCR Master Mix (Applied Biosystems) according to the manufacturer’s instructions and data were normalized by the level of GAPDH or Actin expression in each individual sample. 2^-ΔΔCt^ method was used to calculate relative expression changes. With the help of dissociation curve analysis and the sequencing of PCR products, pairs of specific primers of each cDNAs were designed and selected, without any primer dimers or unspecific amplification detected. The sequences of the primers for Quantitative real-time RT-PCR were in the following table.

| RT-PCR primers | | |
| --- | --- | --- |
| *hIFNβ* | 5'-CTAACTGCAACCTTTCGAAGC | 5'-CTAGTGTCCTTTCATATGCAG |
| *hIP10* | 5'-GGAACCTCCAGTCTCAGCACC | 5'-GGCAGTGGAAGTCCATGAAGTAA |
| *hISG15* | 5'-CTTTGCCAGTACAGGAGCTTG | 5'-GCAGATTCATGAACACGGTGC |
| *hIFI56* | 5'-GACTGTGAGGAAGGATGGGC | 5'-TAGGCTGCCCTTTTGTAGCC |
| *hGAPDH* | 5'-GCTGAGTACGTCGTGGAGTCC | 5'-CCACCCTGTTGCTGTAGCCAAAT |
| *mIfnβ* | 5'-GAGGAAAGATTGACGTGGGAG | 5'-CACCCTCCAGTAATAGCTCTTCAAG |
| *mIp10* | 5'-ATGACGGGCCAGTGAGAATG | 5'-CCCTTGGGAAGATGGTGGTT |
| *mIsg15* | 5'-TCTGACTGTGAGAGCAAGCA | 5'-TGGGGCTTTAGGCCATACTC |
| *mIfi56* | 5'-GTCAAGGCAGGTTTCTGAGGA | 5'-CTTGGCGATAGGCTACGACT |
| *mGapdh* | 5'-TGATGGGTGTGAACCACGAG | 5'-TAGGGCCTCTCTTGCTCAGT |

| QRT-PCR primers | | |
| --- | --- | --- |
| *hIFNβ* | 5'- AGGACAGGATGAACTTTGAC | 5'- TGATAGACATTAGCCAGGAG |
| *hGAPDH* | 5'- ATGACATCAAGAAGGTGGTG | 5'- CATACCAGGAAATGAGCTTG |
| *mIfnβ* | 5'- CACAGCCCTCTCCATCAACT | 5'- TCCCACGTCAATCTTTCCTC |
| *mActin* | 5'- TGACGTTGACATCCGTAAAGAC | 5'- AAGGGTGTAAAACGCAGCTC |

**Type I-IFN bioassay**

Type I-IFN activity was measured as previously described [2], with reference to a recombinant human or mouse IFNβ (from R&D Systems) standard using a 2fTGH cell for human and L929 for mouse (1×10^5^ cells/ml) stably transfected with an IFN-sensitive (ISRE) luciferase construct. Data are represented as mean ± SD.

**Luciferase reporter assay**

293T cells (1×10^5^) were seeded in 24-well plates and transfected the following day by standard calcium phosphate precipitation method. Reporter assay was performed as previously described [1]. Dual-Luciferase Reporter Assay System (Promega) was used. Data are represented as mean ± SD. pNifty-Luc (Invitrogen), an NF-kB-dependent E-selectin-luc reporter plasmid, was purchased as indicated. Promoter region -155-1 of mouse IFNβ was cloned into pGL3-Basic vector (Promega). All constructs were verified by sequencing.

**Primers**

| Primers for sgRNA | | |
| --- | --- | --- |
| traf2 sg1 | 5’-ACCGGCAGCTAGCGTGACCCCCCC | 5’-AAACGGGGGGGTCACGCTAGCTGC |
| traf2 sg2 | 5’-ACCGCCTGCGGAGGACGTTTCTGC | 5’-AAACGCAGAAACGTCCTCCGCAGG |
| traf3 sg1 | 5’-ACCGAGCCCGAAGCAGACCGAGTG | 5’-AAACCACTCGGTCTGCTTCGGGCT |
| traf3 sg2 | 5’-ACCGCCGCTTCTGCGAGAGCTGCA | 5’-AAACTGCAGCTCTCGCAGAAGCGG |
| traf5 sg1 | 5’-ACCGTATACTGGGCTCAAAGTCCA | 5’-AAACTGGACTTTGAGCCCAGTATA |
| traf5 sg2 | 5’-ACCGCTTCCAACCGCTCCACAAAC | 5’-AAACGTTTGTGGAGCGGTTGGAAG |
| traf6 sg1 | 5’-ACCGCTAAACTGTGAAAACAGCTG | 5’-AAACCAGCTGTTTTCACAGTTTAG |
| traf6 sg2 | 5’-ACCGTCACAAGAAACCTGTCTCCT | 5’-AAACAGGAGACAGGTTTCTTGTGA |
| mavs | 5’-ACCGCTGTGAGCTAGTTGATCTCG | 5’-AAACCGAGATCAACTAGCTCACAG |
| nemo | 5’-ACCGCTGCACCTGCCTTCAGAACA | 5’-AAACTGTTCTGAAGGCAGGTGCAG |
| tank | 5’-ACCGAGCCTTCCGGCAGGCATGCA | 5’-AAACTGCATGCCTGCCGGAAGGCT |
| sintbad | 5’-ACCGCGACATCAGCATCCTGACGC | 5’-AAACGCGTCAGGATGCTGATGTCG |
| Ikkα | 5’-ACCGGTACCAAAAACAGAGAACGA | 5’-AAACTCGTTCTCTGTTTTTGGTAC |
| Ikkβ | 5’-ACCGTCAGCCCCCGGAACCGAGAG | 5’-AAACCTCTCGGTTCCGGGGGCTGA |

| Primers for NEMO knock-out MEF genotyping | |
| --- | --- |
| P1 | 5’-TCCGGTTCTGTCGGAGCGGTC |
| P2 | 5’-ACCCACTCGTGCACCCAACTG |
| P3 | 5’-TGAGGGACCAGGACCCTTTGC |
| P4 | 5’-GTCAGGAGGAGCCTGGGATAC |

1. Sun W, Li Y, Chen L, Chen H, You F, Zhou X, et al. ERIS, an endoplasmic reticulum IFN stimulator, activates innate immune signaling through dimerization. Proceedings of the National Academy of Sciences of the United States of America. 2009;106(21):8653-8. Epub 2009/05/13. doi: 10.1073/pnas.0900850106

0900850106 [pii]. PubMed PMID: 19433799; PubMed Central PMCID: PMC2689030.

2. Jiang Z, Georgel P, Du X, Shamel L, Sovath S, Mudd S, et al. CD14 is required for MyD88-independent LPS signaling. Nature immunology. 2005;6(6):565-70. Epub 2005/05/17. doi: ni1207 [pii]

10.1038/ni1207. PubMed PMID: 15895089.
