# Supplementary material for: MAVS activates TBK1 and IKKε through TRAFs in NEMO dependent and independent manner
Source: PLoS Pathog. 2017 Nov 10;13(11):e1006720. doi: 10.1371/journal.ppat.1006720 (PMC5699845; doi:10.1371/journal.ppat.1006720)
Supplement: S1 Fig — (A) to (D) The deletion of TANK/NAP1/SINTBAD in TANK−/−NAP1−/−SINTBAD−/− 293T cells was detected with Genotyping (A, B, C) and Western blot analysis (D). (E) to (G) WT and TANK−/−NAP1−/−SINTBAD−/− 293T cells were infected with SeV for the indicated times. Cells were analyzed by Western blot to detect the phosphorylation and expression of the indicated proteins (E), or RT-PCR analysis of the indicated gene induction (F), bioassay analysis of type I-IFN production (G). Data from (G) represent mean ± SD. Similar results were obtained in 3 independent experiments. (PDF) [file ppat.1006720.s001.pdf]

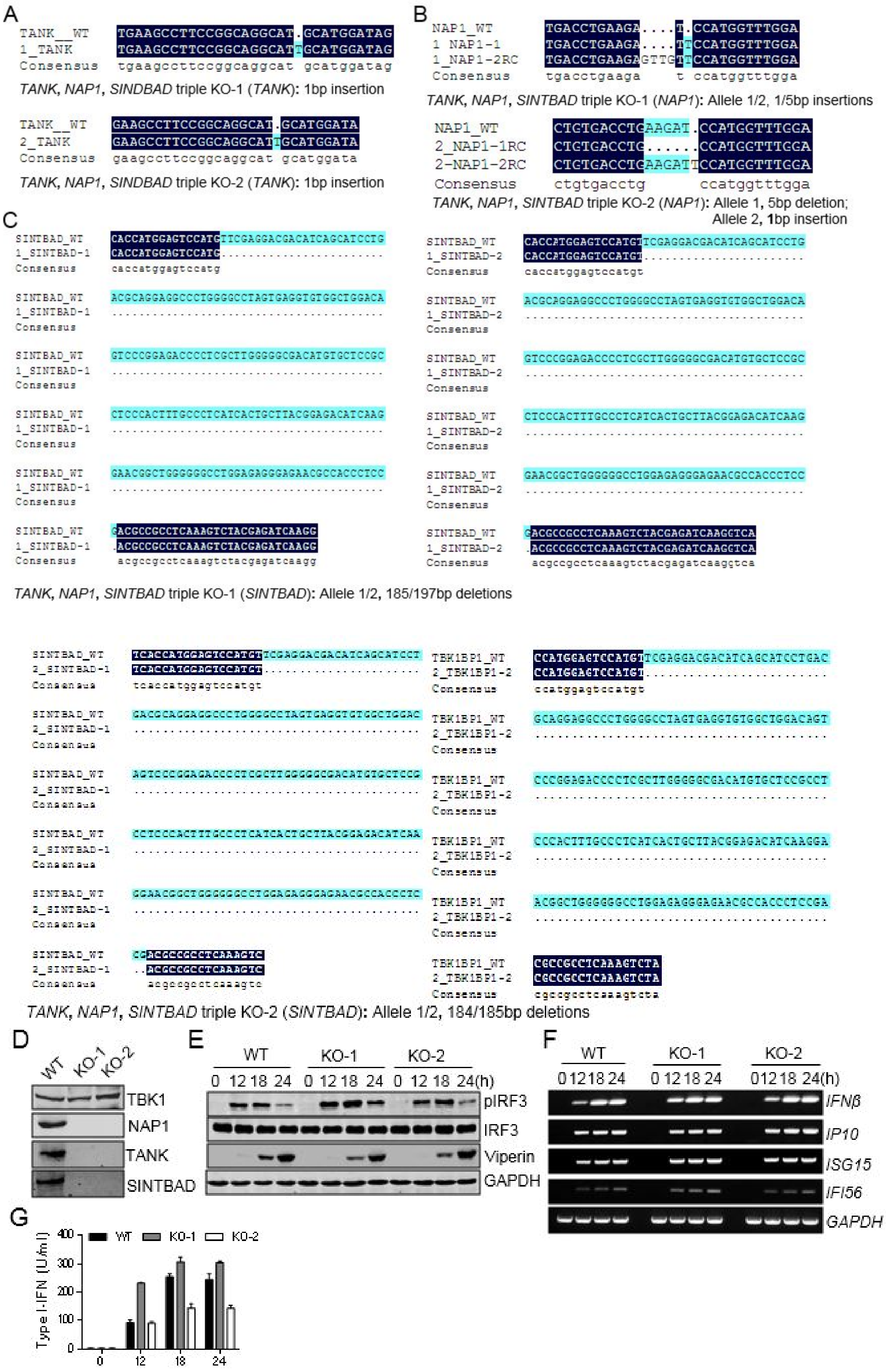

Fig S1. TBK1-binding proteins TANK/NAP1/SINTBAD are not required for RLR-MAVS pathway
